# Computationally designed sensors detect endogenous Ras activity and signaling effectors at subcellular resolution

**DOI:** 10.1038/s41587-023-02107-w

**Published:** 2024-01-25

**Authors:** Jason Z. Zhang, William H. Nguyen, Nathan Greenwood, John C. Rose, Shao-En Ong, Dustin J. Maly, David Baker

**Affiliations:** 1 Department of Biochemistry, University of Washington, Seattle, WA USA; 2 Institute for Protein Design, University of Washington, Seattle, WA USA; 3 Howard Hughes Medical Institute, University of Washington, Seattle, WA USA; 4 Department of Dermatology, Stanford University School of Medicine, Stanford, CA USA; 5 Department of Pharmacology, University of Washington, Seattle, WA USA; 6 Department of Chemistry, University of Washington, Seattle, WA USA

**Keywords:** Protein design, Fluorescent proteins, Cancer imaging

## Abstract

The utility of genetically encoded biosensors for sensing the activity of signaling proteins has been hampered by a lack of strategies for matching sensor sensitivity to the physiological concentration range of the target. Here we used computational protein design to generate intracellular sensors of Ras activity (LOCKR-based Sensor for Ras activity (Ras-LOCKR-S)) and proximity labelers of the Ras signaling environment (LOCKR-based, Ras activity-dependent Proximity Labeler (Ras-LOCKR-PL)). These tools allow the detection of endogenous Ras activity and labeling of the surrounding environment at subcellular resolution. Using these sensors in human cancer cell lines, we identified Ras-interacting proteins in oncogenic EML4-Alk granules and found that Src-Associated in Mitosis 68-kDa (SAM68) protein specifically enhances Ras activity in the granules. The ability to subcellularly localize endogenous Ras activity should deepen our understanding of Ras function in health and disease and may suggest potential therapeutic strategies.

## Abstract (web summary)

Computationally designed sensors map the activity and signaling environment of Ras.

## Main

**Fig. 1:**
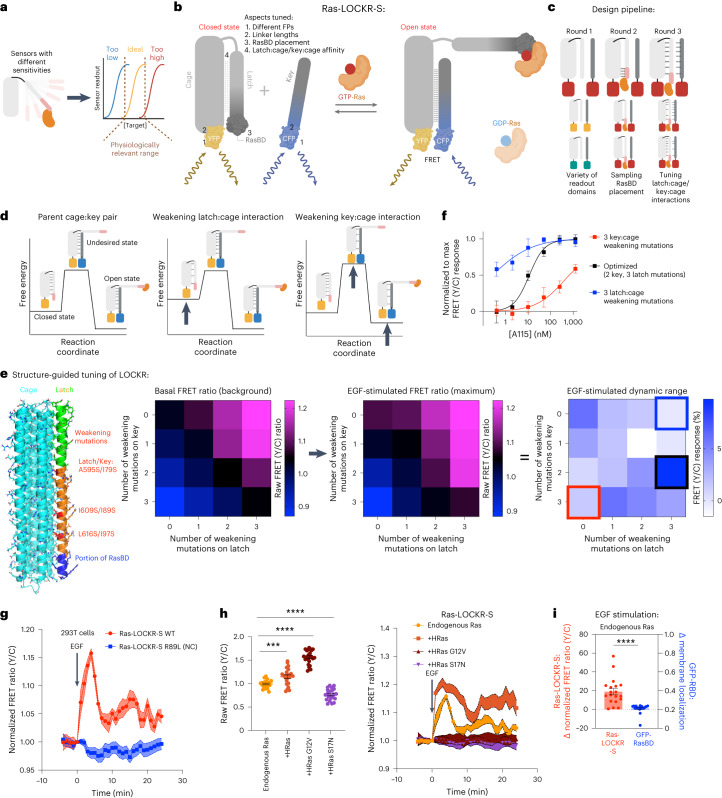
A LOCKR-based sensor (Ras-LOCKR-S) measures endogenous Ras activity. **a**, Schematic depicting the difficulty in matching sensor sensitivity to the biologically relevant concentration range. **b**, Ras-LOCKR-S optimization. **c**, Design pipeline. **d**, Avenues for tuning Ras-LOCKR-S switching energetics. **e**, Experimental tuning of Ras-LOCKR-S. Left, predicted structure of Ras-LOCKR-S with mutations highlighted. Right, heat maps of Ras-LOCKR-S with latch:cage or key:cage weakening mutations. FRET ratios (yellow/cyan) before (background) and after (maximum) 100 ng ml^−1^ EGF stimulation (*n* = 14 cells per condition) in 293T cells transiently expressing Ras-LOCKR-S. Dynamic range equals maximum FRET ratio (EGF) minus basal FRET ratio. The colored boxes in EGF-stimulated dynamic range heat map correspond to the constructs tested in **f**. **f**, Normalized to maximum FRET responses (calculated by setting the lowest and highest FRET ratios in each dataset to 0 and 1, respectively) in CIAR-PM-293 cells transiently expressing Ras-LOCKR-S mutants and treated with varying doses of A115 (*n* = 12–15 cells per A115 concentration). **g**, Normalized FRET ratio changes in 293T cells transiently expressing WT or NC Ras-LOCKR-S and stimulated with 100 ng ml^−1^ EGF (*n* = 10 cells per condition). **h**, Starting raw FRET ratios (left) and average normalized FRET ratio changes (right) in Ras-LOCKR-S-expressing 293T cells co-expressing WT HRas, HRas G12V, HRas S17N or no exogenous Ras (left: *n* = 10 cells per condition; right: *n* = 23 cells per condition). **i**, Comparison of Ras-LOCKR-S to GFP-RasBD in response to 100 ng ml^−1^ EGF in 293T cells (*n* = 19 cells). Solid lines indicate representative average timecourse, with error bars representing s.e.m. ****P = 3.5 × 10^−5^. Bar graphs represent mean ± s.e.m. ****P < 0.0001 and ***P < 0.001, ordinary one-way ANOVA. All LOCKR-S candidates and associated experimental results are listed in Supplementary Table 1. WT, wild-type, NC, negative control.

Precise regulation of Ras activity is critical for normal cell function, and mutation of Ras occurs frequently in cancers^1^. Ras GTPases switch between GDP-bound (inactive) and GTP-bound (active) states, which are dynamically regulated by guanine exchange factors (GEFs) and Ras GTPase-activating proteins (GAPs) that promote the active and inactive states, respectively. Ras-GTP levels change rapidly in response to growth factor activation of receptor tyrosine kinases (RTKs) to promote mitogen-activated protein kinase (MAPK) signaling. Ras is associated with multiple intracellular organelles, but its activity and function in different subcellular regions is not well defined^2–4^. Ras activation was thought to require membranes, but membrane-less granules formed by oncoprotein RTK fusions, such as EML4-Alk, can have associated Ras activity^5^; the mechanism of Ras activation inside EML4-Alk granules is unclear. Overall, despite decades of study, there are still many open questions about the spatiotemporal activity of Ras due to the lack of tools for detecting endogenous Ras activity. Although optogenetic and chemogenetic systems for directly activating Ras have been developed, such as Chemically Inducible Activator of Ras (CIAR)^6^, there are no sensors that can measure real-time, subcellular activity of endogenous levels of Ras-GTP^2,4,7–10^ (Fig. 1a), which are in the nanomolar range^11^. Most biosensors are based on native protein-binding domains^12^, but these are limited in number and often cannot be engineered without reducing affinity or specificity, thus limiting opportunities for tuning sensor dynamic range^10^ (Fig. 1a).

We reasoned that the computational protein design could provide greater control over sensor switching energetics and overcome these limitations. We sought to generate both fluorescent activity sensors based on Förster resonance energy transfer (FRET) and proximity labelers of the environment of activated Ras. Using these tools, we identified Ras activity in EML4-Alk granules that was associated with Src-Associated in Mitosis 68-kDa (SAM68) protein, providing insight into mechanisms of Ras activation in membrane-less granules.

## Results

### Computational design of LOCKR-based Ras activity sensors

**Extended Data Fig. 1:**
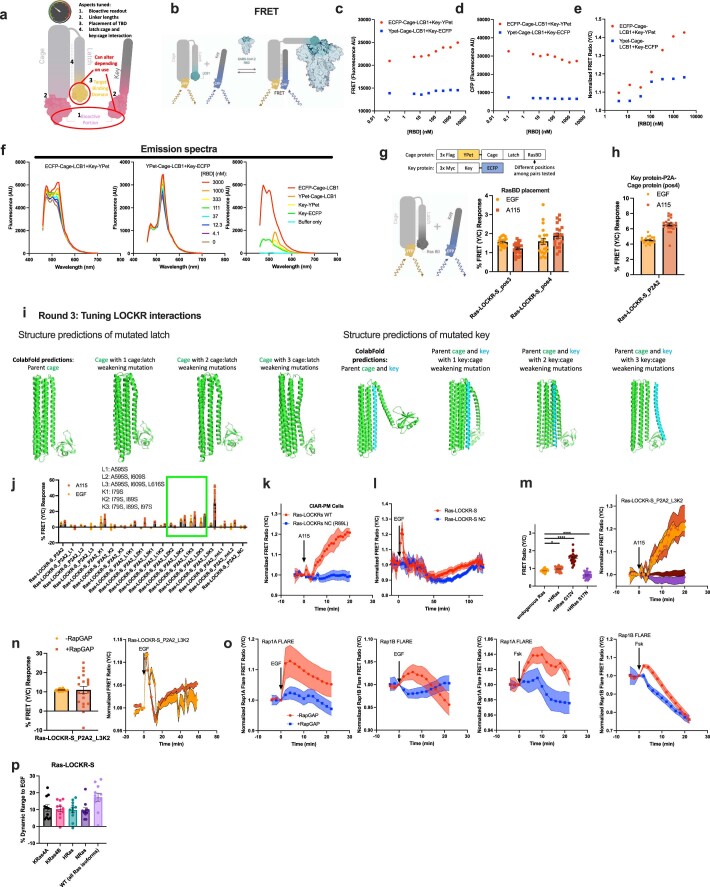
Design and characterization of Ras-LOCKR-S to track compartmentalized endogenous Ras activity dynamically and specifically. **a**, Schematic describing the different aspects of Ras-LOCKR-S that were tuned. **b–e**, FRET-based LOCKR sensors modifying lucCageRBD, which senses RBD of SARS-CoV-2 spike protein via binding to the *de novo* protein binder LCB1, (**b**) with CFP and YFP as FRET donor and acceptor, respectively. Two placements of the FPs were tested *in vitro* for yellow/cyan FRET fluorescence (**c**), CFP fluorescence (**d**), and normalized yellow/cyan FRET ratio changes (normalized to no RBD) (**e**) with a range of RBD concentrations ([Cage]=[Key]=1 mM). **f**, FRET spectra (excitation wavelength: ECFP+YPet, ECFP only and buffer only=450 nm, YPet only=510 nm) of the two Key-Cage pairs against a range of RBD concentrations ([Cage]=[Key]=1 mM) (left and middle) and the Key or Cage proteins alone (right). **g**, (top) Domain structures shown. (bottom) Various FRET-based Ras-LOCKR-S candidates with different RasBD grafting positions on latch using Rosetta-based GraftSwitchMover (see Methods) were tested in 293T cells (100 ng/mL EGF stimulation) and CIAR-PM-293 (250 nM A115 stimulation) cells with percent FRET ratio changes reported (n = 20 cells per condition). Only YFP-tagged Cage and CFP-tagged Key were tested as YFP-tagged Key did not show fluorescence. **h**, RasBD placements identified in Round 1 were used in unimolecular (only Cage, no Key) without linker, with linker, and bimolecular (with P2A sequence) formats. One of the bimolecular designs showed consistent responses to EGF and A115 (n = 20 cells per condition). **i**, AlphaFold structure predictions of Ras-LOCKR-S Cage only (left) or Key:Cage complex (right) with the indicated mutations. **j**, Point mutations to weaken latch:cage or key:cage interactions (the N-terminal half of latch and key are similar) were identified via structure predictions and implemented in the bimolecular Ras-LOCKR-S candidate identified in **h**. The Ras-LOCKR-S candidates that displayed consistent FRET ratio changes to EGF and A115 are boxed in green and were further validated in subsequent experiments (n = 20 cells per condition). **k**, Ras-LOCKR-S negative control (NC, RasBD^R89L^) showed negligible FRET ratio changes (n = 10 cells per condition). **l**, Long-term imaging of Ras-LOCKR-S WT and NC in 293T cells stimulated with 100 ng/mL EGF (n = 15 cells per condition). **m**, CIAR-PM-293 cells expressing Ras-LOCKR-S_P2A2_L3K2 and co-expressing either WT HRas, constitutively active HRas (HRas^G12V^), or dominant negative HRas (HRas^S17N^) were stimulated with 250 nM A115. FRET ratio changes (right, n = 11 cells per condition) and starting FRET ratios (left, n = 25 cells per condition) are reported. **n**, Ras-LOCKR-S_P2A2_L3K2 was tested for selectivity for Ras in 293T cells expressing RapGAP and stimulated with EGF (left, n = 20 cells per condition) (right, n = 13 cells per condition). Statistics: +HRas *p = 0.01, +HRas G12V ****p = 3.9 × 10^−12^, +HRas S17N ****p = 3.4 × 10^−8^. **o**, Testing of RapGAP^50^ in 293T cells using Rap1A and Rap1B FLARE activity reporters^19^. FRET ratio changes of 293T cells expressing either Rap1A FLARE or Rap1B FLARE with and without RapGAP co-expressionin response to stimulation with either EGF (left) or 50 *μ*M Forskolin (Fsk) (n = 25 cells per condition for EGF experiments, 27 cells per condition for Fsk experiments). **p**, FRET ratio dynamic range of Ras-LOCKR-S expressed in WT MEF cells or MEF cells expressing one major isoform of Ras stimulated with 100 ng/mL EGF (n = 11 cells per condition). See Supplementary Table 1 for domain structures and sequences. For all graphs, solid lines indicate representative average timecourses of FRET ratio changes with error bars representing standard error mean (s.e.m.). Bar graphs represent mean ± s.e.m. ****p < 0.0001, ordinary one-way ANOVA.

We sought to design Ras sensors with the following properties: (1) sensitivity to endogenous levels of active Ras, (2) compatibility with live-cell imaging, (3) ability to report activity in different subcellular regions and (4) single-cell resolution. We reasoned that the designed LOCKR switch system^13,14^ was well suited to sense active Ras (Extended Data Fig. 1a) due to its generalizability, orthogonality and tunability. LOCKR is a two-component system consisting of (1) a Cage protein that contains a ‘cage’ domain, a ‘latch’ domain that intramolecularly interacts with the cage and embeds a target binding domain (TBD) and one portion of a split molecular readout and (2) a Key protein that contains a cage domain-binding ‘key’ domain and the other portion of the split molecular readout. In the absence of target, the latch of the Cage is closed, blocking interaction of the key with the cage, resulting in low readout signal. Interaction of a target protein with the TBD opens the latch, allowing the key to bind the cage and reconstitution of the split readout module^13^ (Fig. 1b).

We designed Ras LOCKR sensors (Ras-LOCKR-S) (Fig. 1c) in three steps. Because the low spatial resolution of the luminescent readout of previously developed LOCKR-based sensors is not optimal for subcellular reporting^12^, we first determined the compatibility of the LOCKR system with FRET^15^-based readout (Supplementary Table 1). To do this, we converted the readout of a LOCKR-based sensor for the receptor-binding domain (RBD) of SARS-CoV-2 lucCageRBD^14,16^ to a FRET readout by placing CFP and YFP at the termini of Key and Cage (Extended Data Fig. 1a–f). RBD addition increased FRET ratios (YFP/CFP) by up to 40% (Extended Data Fig. 1e) for all FP placements, indicating the feasibility of FRET as a readout for LOCKR-based sensors.

Next, we embedded the Ras-binding domain (RasBD) from Raf1 (ref. ^17^), which is selective for Ras-GTP, within the latch of our FRET-based LOCKR system to sense Ras-GTP^17^. To determine the placement of RasBD, we used Rosetta-based GraftSwitchMover^13^ to graft the RasBD onto the latch, varying the register of the interaction of the embedded RasBD with the cage (Fig. 1b). This identified seven possible RasBD placements, which we tested in 293T cells for their responsiveness to epidermal growth factor (EGF)-mediated increases in Ras-GTP levels. The two candidates with a detectable FRET ratio increase (1.5%) in response to EGF stimulation also displayed FRET ratio increases upon activation of a PM-localized CIAR in 293 cells (CIAR-PM-293) with A115 (ref. ^6^) (Extended Data Fig. 1g). The highest dynamic range candidate (Ras-LOCKR-S_pos4) from RasBD placement testing was further optimized by ensuring similar expression of Key and Cage using a P2A sequence (Extended Data Fig. 1h and Supplementary Table 1).

Finally, we tuned the free energies of the three states of LOCKR-S^14^ (closed, open and an undesired false-positive state where Key binds to Cage without target binding (Fig. 1d)) to adjust the sensor to be maximally sensitive to physiologically relevant Ras-GTP concentrations (Fig. 1a). To do so, we used AlphaFold^18^ to identify Key and Cage mutations that modulate the probability of key:cage and latch:cage interactions (Extended Data Fig. 1i) to alter the binding free energies of LOCKR-S’s three thermodynamic states (Fig. 1d). We found that, as expected, key:cage weakening mutations decreased background and EGF-stimulated FRET ratios, whereas latch:cage weakening mutations increased background and maximum FRET ratios (Fig. 1e; the latter promote key:cage interactions, which generate the FRET signal). We evaluated the responses of candidates with variable key:cage and latch:cage interactions to different levels of Ras-GTP levels by titrating A115 doses in CIAR-PM-293s. We found that the Ras-LOCKR-S candidate possessing a Key with reduced affinity for the Cage (Fig. 1f, red line) responded only to high Ras-GTP levels. Meanwhile, the Ras-LOCKR-S candidate with a weakened latch interaction with the cage (Fig. 1f, blue line) had high background FRET at low Ras-GTP levels that minimally increased at higher A115 concentrations (Fig. 1f). The Ras-LOCKR-S_P2A2_L3K2 (three mutations in latch and two mutations in key) construct showed the highest FRET ratio increase (10%) upon EGF stimulation and robust FRET ratio increases (10%) upon A115 treatment of CIAR-PM-293s (Fig. 1e and Extended Data Fig. 1j–k). Ras-LOCKR-S_P2A2_L3K2 had a wide range of FRET responses across A115 doses (Fig. 1f, black line, ‘optimized’) and, hence, is well suited for sensing physiologically relevant concentrations of endogenous Ras-GTP; this Ras-LOCKR-S construct is used throughout the rest of the paper.

We characterized the temporal response of Ras-LOCKR-S (untargeted, cytosol localization) to EGF stimulation. Consistent with the well-characterized dynamics of Ras-GTP levels resulting from EGF stimulation, we observed a rapid and transient FRET ratio increase (Fig. 1g), demonstrating Ras-LOCKR-S’s rapid sensing and reversibility in response to variable Ras-GTP levels. Ras-LOCKR-S can be imaged over extended (2 h) timecourses, which should be useful for following longer-term physiological processes (Extended Data Fig. 1l). Ras-LOCKR-S exhibited sustained FRET ratio increases in response to treating CIAR-PM-293s with A115 (Extended Data Fig. 1k), reflecting the dynamics previously observed with Ras-GTP pulldowns^6^. Under the same conditions, we found no changes in FRET ratios for a negative control (NC) Ras-LOCKR-S construct that contains a mutation (RasBD^R89L^) that abrogates Ras-GTP binding^17^ (Extended Data Fig. 1k,l). Further confirming Ras-LOCKR-S’s Ras-GTP-sensing ability, we observed that co-expression of a constitutively active mutant of Ras (HRas^G12V^) or a dominant negative mutant of Ras (HRas^S17N^) increased and decreased raw FRET ratios, respectively (Fig. 1h). HRas^S17N^ co-expression also eliminated FRET ratio changes after EGF stimulation or A115 activation of CIAR-PM-293s (Fig. 1h and Extended Data Fig. 1m). Co-expression of a GAP for Rap GTPase (structurally similar to Ras) minimally influenced Ras-LOCKR’s response to EGF stimulation (Extended Data Fig. 1n) but did abrogate a Rap1 biosensor’s (Rap1 FLARE)^19^ response (Extended Data Fig. 1o). Ras-LOCKR-S more robustly reported endogenous Ras activation from EGF stimulation (Fig. 1i) than GFP-RasBD^4^, which is commonly used to measure Ras activity. Overall, Ras-LOCKR-S is a quantitative sensor capable of real-time reporting of endogenous Ras activity across all Ras isoforms (Extended Data Fig. 1p).

### Ras-LOCKR-S detects endogenous Ras activity

**Fig. 2:**
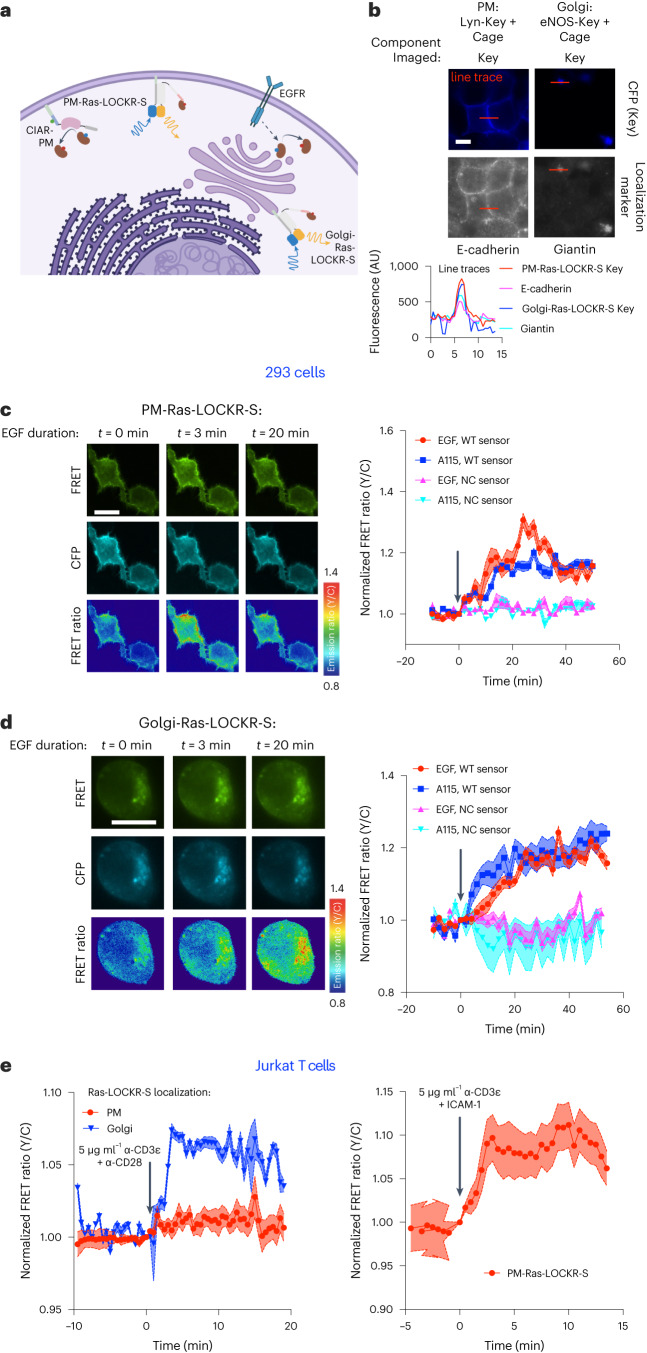
Ras-LOCKR-S can report subcellular endogenous Ras activity in multiple cell types. **a**, Schematic of subcellularly targeted Ras-LOCKR-S, EGF receptor (EGFR) and CIAR at the PM (CIAR-PM). Localization sequences: PM: N-terminus of Lyn; Golgi: N-terminus of eNOS. **b**, Top, representative images of 293T cells transfected with localized Ras-LOCKR-S (Key with localization sequence, Cage untargeted) and stained for localization markers. Bottom, line trace comparison of CFP signal from Ras-LOCKR-S Key and localization marker. Scale bar, 5 μm. **c**,**d**, CIAR-PM-293 cells expressing Ras-LOCKR-S WT or NC localized to PM (**c**) or Golgi (**d**) were stimulated with either 100 ng ml^−1^ EGF or 250 nM A115. Left, representative epifluorescence images using ×60 objective (CFP channel from Key, FRET channel and pseudocolored raw FRET ratio) from three biologically independent experiments. Right, normalized FRET ratio changes (*n* = 16 cells per condition for WT PM and NC Golgi experiments, *n* = 20 cells per condition for NC PM experiments and *n* = 15 cells per condition for WT Golgi experiments). Scale bars, 10 μm. **e**, Average normalized FRET ratio responses of Jurkat T cells expressing Ras-LOCKR-S localized to Golgi or PM stimulated with either 5 μg ml^−1^ of α-CD3*ε* + *α*-CD28 (left) or 5 μg ml^−1^ of *α*-CD3*ε* + intracellular adhesion molecule 1 (ICAM-1; right) coated on the plate (*n* = at least 10 cells per condition). Solid lines in **c**,**d** indicate a representative average timecourse from three biologically independent experiments, with error bars representing s.e.m. Solid lines in **e** indicate average timecourses of FRET ratio changes from all cells combined from three experiments, with error bars representing s.e.m. WT, wild-type, NC, negative control.

**Extended Data Fig. 2:**
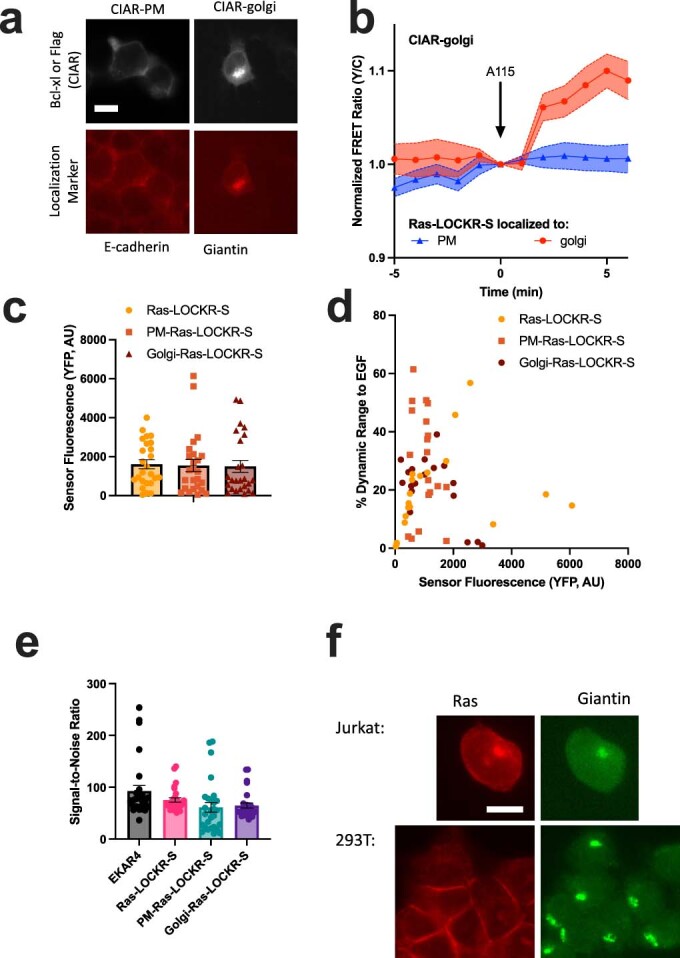
Characterization of localized Ras-LOCKR-S and CIAR in 293 T and Jurkat cells. **a**, CIAR localized to PM (KRas4a CAAX) or Golgi (Giantin^3131–3259^)^51^ is stably expressed in 293-TRex cells, which were immunostained for CIAR via Bcl-xL (CIAR-PM) or Flag tag (CIAR-Golgi) and their respective localization markers with representative epifluorescence images shown from 3 biologically independent experiments. **b**, FRET ratio changes after A115 addition to cells stably expressing Golgi-localized CIAR (CIAR-Golgi) and transfected with subcellularly localized Ras-LOCKR-S (n = 9 cells per condition). **c**, Average localized Ras-LOCKR-S (Cage, YFP) fluorescence (n = 25 cells per condition). **d**, Scatterplot of sensor fluorescence (Cage, YFP) compared to % dynamic range to EGF. **e**, Comparison of the signal-to-noise ratios for EKAR4, untargeted Ras-LOCKR-S, PM-Ras-LOCKR-S, and Golgi-Ras-LOCKR-S in 293T cells stimulated with 100 ng/mL EGF (n = 28 cells per condition). **f**, Representative epifluorescence images from 3 biologically independent experiments of Jurkat T cells and 293T cells immunostained for Ras and giantin (Golgi marker). Solid lines indicate representative average timecourses of FRET ratio changes with error bars representing standard error mean (s.e.m.). Bar graphs represent mean ± s.e.m. Scale bars = 10 *μ*m.

We developed subcellularly localized Ras-LOCKR-S to measure endogenous Ras activity in different locations (Fig. 2a) by restricting the Key to the PM or Golgi using localization tags^20–23^ (Fig. 2b). Restriction of Key localization resulted in FRET ratio increases primarily in the target subcellular regions in response to EGF stimulation (Fig. 2c,d). We next explored if Golgi-localized Ras-LOCKR-S (Golgi-Ras-LOCKR-S) could detect endogenous Ras-GTP generated at this subcellular location (Extended Data Fig. 2a) by a Golgi-localized CIAR (CIAR-Golgi) construct. Treatment of CIAR-Golgi-expressing 293s with A115 led to FRET ratio increases for Golgi-Ras-LOCKR-S but not for PM-Ras-LOCKR-S (Extended Data Fig. 2b), confirming that these localized Ras sensors are reporting compartment-specific activities. Both EGF and A115 treatment of CIAR-PM-293s led to FRET ratio increases for Golgi-Ras-LOCKR-S (Fig. 2d), suggesting that endogenous Ras signaling occurs at endomembranes probably due to Ras trafficking^24^. Untargeted and subcellularly targeted Ras-LOCKR-S sensors expressed at similar levels (Extended Data Fig. 2c) and sensor expression levels did not correlate with their EGF-stimulated FRET ratio increases (Extended Data Fig. 2d), suggesting that sensor expression levels do not determine dynamic range. The signal-to-noise ratios for untargeted and subcellularly targeted Ras-LOCKR-S sensors were similar to the widely used EKAR4 sensor (Extended Data Fig. 2e).

Next, we explored the utility of Ras-LOCKR-S for reporting subcellular endogenous Ras-GTP levels in other cell types. Compared to HEK293Ts, Ras in Jurkat T cells is highly enriched at the Golgi (Extended Data Fig. 2f)^25,26^. We found that T cell receptor (TCR)-activated Jurkat cells displayed FRET ratio increases for Golgi-Ras-LOCKR-S but not PM-Ras-LOCKR-S (Fig. 2e), demonstrating that TCR activation leads to endogenous Ras activity at the Golgi. In contrast, co-stimulation of TCR and lymphocyte function-associated antigen 1 (LFA-1) led to FRET ratio increases for PM-Ras-LOCKR-S (Fig. 2e). These observations are consistent with previous studies showing that TCR stimulation activates overexpressed NRas, which is primarily localized to the Golgi in Jurkat cells, and that co-stimulation of the TCR and LFA-1 is required for Ras signaling at the PM^25,26^. Thus, Ras-LOCKR-S can report endogenous Ras activity in multiple cell types, and targeted Ras-LOCKR-S detects compartment-specific Ras activity.

### Design of Ras activity-dependent proximity labelers

**Fig. 3:**
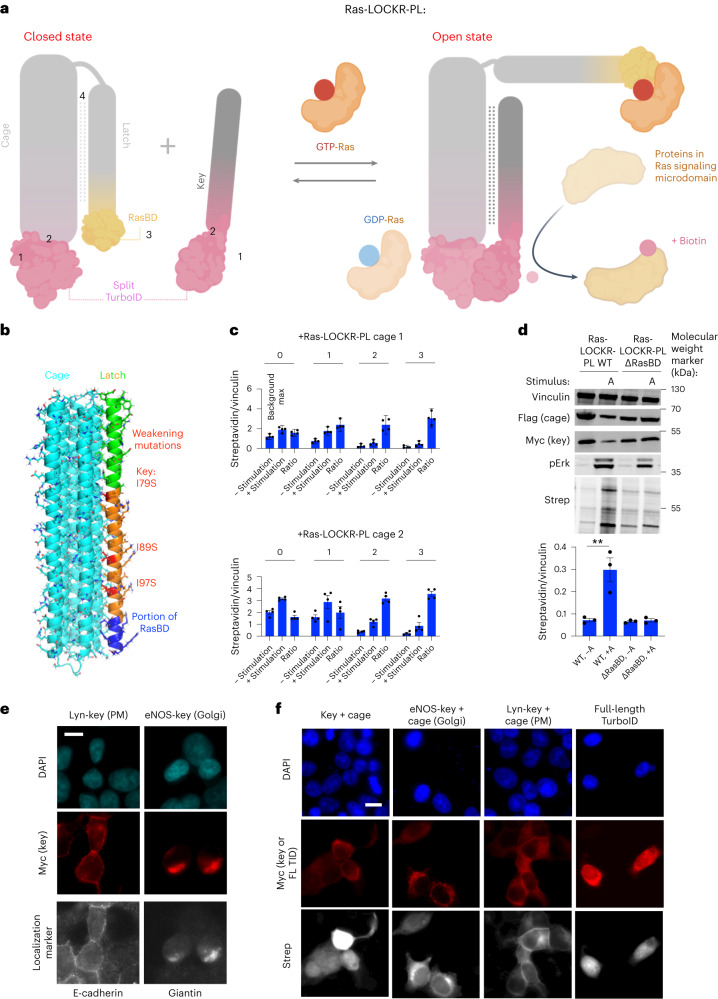
De novo designed Ras-dependent LOCKR-based proximity labeler (Ras-LOCKR-PL) identifies Ras targets in signaling microdomains. **a**, Schematic of optimized Ras-LOCKR-PL consisting of split TurboID tethered to Cage and Key; GTP-Ras binds to RasBD, promoting reconstitution of functional TurboID and biotinylation of neighboring proteins. As in Fig. 1b, numbers correspond to regions that were optimzied during the development of Ras-LOCKR-PL. **b**, Predicted structure of Ras-LOCKR-PL with mutations highlighted. **c**, Bar graph of biotinylation levels of CIAR-PM-293 cells, which were transfected with Ras-LOCKR-PL candidates, and 500 μM biotin was added for 16 h without (−stimulation) or with (+stimulation) 250 nM A115 (*n* = 4 experiments per condition). Ratio is +stimulation divided by −stimulation. The numbers shown over the bar graphs represent the number of key:cage weakening mutations. **d**, Representative western blots of CIAR-PM-293 cells transfected with Ras-LOCKR-PL WT or a mutant without RasBD (ΔRasBD), and treated with 500 μM biotin with or without 250 nM A115 (labeled ‘A’) for 16 h (*n* = 3 experimental repeats). **e**,**f**, Representative epifluorescence images from three biologically independent experiments. CIAR-PM-293 cells were transfected with subcellularly localized Ras-LOCKR-PL or full-length TurboID expressed, were treated with 500 μM and 250nM A115 for **f** and underwent immunostaining with antibodies for established localization markers (**e**) or fluorescent dye-conjugated streptavidin (**f**). Bar graphs represent mean ± s.e.m. **P < 0.01, unpaired two-tailed Student’s *t*-test. Scale bars, 10 μm. All LOCKR-PL candidates and associated experimental results are listed in Supplementary Table 2.

To enable identification of upstream Ras activators and downstream effectors in different cellular compartments, we developed a Ras-GTP-activated proximity labeler also based on LOCKR (LOCKR-based, Ras activity-dependent Proximity Labeler (Ras-LOCKR-PL)) (Fig. 3a and Supplementary Table 2). We reasoned that a Ras-GTP-activated proximity labeler could be generated by replacing the FPs in Ras-LOCKR-S with split biotin ligases (Fig. 1c), allowing functional proximity labeler assembly upon Ras-GTP binding to RasBD (Fig. 3a).

**Extended Data Fig. 3:**
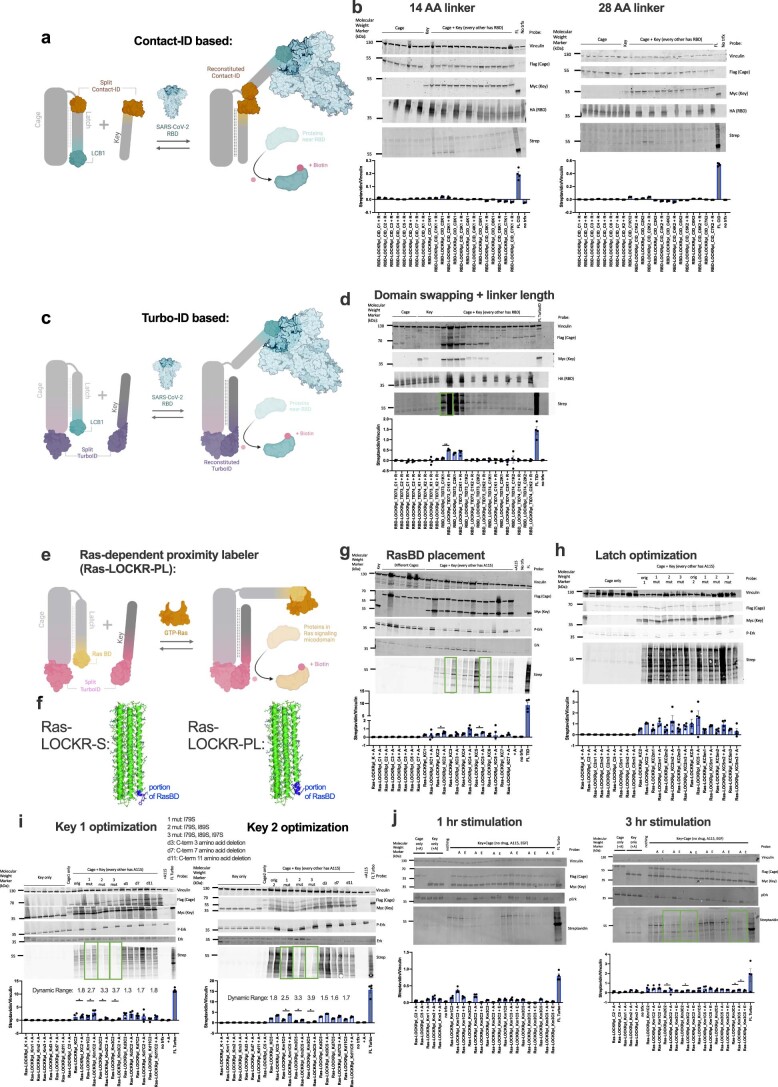
Development of Ras-LOCKR-PL. **a, b**, Split ContactID-based LOCKR proximity labelers were tested using similar architecture to lucCageRBD. Schematic in **a** and data shown in **b**. Different grafting positions of the smaller bit of ContactID on the latch using Rosetta-based GraftSwitchMover (see Methods) and different linker lengths between larger bit of ContactID and Key were tested in 293T cells transfected with Flag-tagged Cage, Myc-tagged Key, full length ContactID (FL CID), and HA-tagged RBD (receptor binding domain of SARS-CoV-2 spike protein) with 5x nuclear exclusion sequence. After 16 h of 500 *μ*M biotin incubation, these cells were subsequently western blotted for the transfected proteins and biotinylation via streptavidin (n = 4 biologically independent experiments per condition). **c**, **d**, 293T cells were transfected with Split TurboID (split site TurboID^73/74^)-based LOCKR Cages, Keys, or Keys with Cages, which were coexpressed with or without RBD, and biotinylation levels were probed following lysis. Biotinylation levels of cells expressing full length TurboID (FL TID) are also shown. Different placements of the split TurboID and linker lengths between split TurboID and Cage/Key were tested with the Ras-LOCKR-PL candidate that led to the highest increase in biotinylation upon RBD expression boxed in green and optimized further (n = 4 biologically independent experiments per condition). Statistics: **p = 0.0058. Schematic in **c** and data shown in d. **e–j**, Testing of Ras-LOCKR-PL candidates was done via western blotting of CIAR-PM-299 cells incubated with 250 nM A115 (labeled A) or 100 ng/mL EGF (labeled E) and 500 *μ*M biotin for either 16 hour (g-i), 1 h (**j**, left), or 3 h (**j**, right) (n = 4 biologically independent experiments per condition). RasBD was grafted onto the latch using GraftSwitchMover (**f**, shows structure predictions) and tested for increases in biotinylation after Ras activation by A115 (FL = full length TurboID) (n = 4 biologically independent experiments per condition). The highest dynamic range Ras-LOCKR-PL candidates are boxed in green (**g**) and were further optimized by either weakening latch:cage interaction by mutating latch (**h**) or weakening key:cage interaction by mutating the Key (2 different Cages tested for left and right) (**i**) (n = 4 biologically independent experiments per condition). Statistics for **g** from left to right: *p = 0.032, *p = 0.025. C-terminal Key truncations were also tested to weaken key:cage interaction (**i**) (n = 4 biologically independent experiments per condition). Statistics for **i** from left to right: *p = 0.042, *p = 0.015, *p = 0.032, *p = 0.044, *p = 0.017, *p = 0.023, *p = 0.027. The highest dynamic range Ras-LOCKR-PL candidates boxed in green (**i**) were tested in CIAR-PM-293 cells treated for shorter times with A115 or EGF (**j**) (n = 4 biologically independent experiments per condition). Statistics for **j** from left to right: *p = 0.021, *p = 0.036, *p = 0.048, *p = 0.018. See Supplementary Table 2 for domain structures and sequences. Bar graphs represent mean ± s.e.m. All statistics are derived from a two-way student t-test.

We first tested whether split versions of the biotin ligases ContactID^27^ and TurboID^28^ are compatible with the LOCKR system by swapping the split luciferase in lucCageRBD^14,16^ with split biotin ligase (RBD-LOCKR-PL). In HEK293T cells expressing RBD, the split ContactID-containing RBD-LOCKR-PL (Extended Data Fig. 3a,b) showed no increase in biotinylation of cellular proteins (probably due to its lower dynamic range^28^), whereas the split TurboID-containing RBD-LOCKR-PL (Extended Data Fig. 3c,d) showed RBD-dependent biotinylation. Thus, we used split TurboID for constructing an optimized Ras-LOCKR-PL. To design Ras-LOCKR-PL, we again used SwitchGraftMover to identify possible RasBD placements on latch. The most promising candidates (RasBD interacting with cage) were tested for Ras-GTP-activated labeling in CIAR-PM-293s treated with A115 for 16 h (Extended Data Fig. 3e,g). We found that an offset of five amino acids relative to the RasBD placement within Ras-LOCKR-S led to the highest dynamic range (100% increase in biotinylation from A115 treatment) (Extended Data Fig. 3f). We tuned the interactions of the latch and key with cage to maximize the dynamic range of the response to A115 treatment of CIAR-PM-293s (Fig. 3b). Mutating the latch to decrease the latch:cage interaction strength increased background but not A115-stimulated signal, resulting in a decreased dynamic range (Extended Data Fig. 3h). Mutations that weaken the key:cage interaction consistently decreased background biotinylation, leading to Ras-LOCKR-PL candidates with an overall increased dynamic range (200–300% increase in biotinylation in response to A115 treatment) (Fig. 3c and Extended Data Fig. 3i).

**Extended Data Fig. 4:**
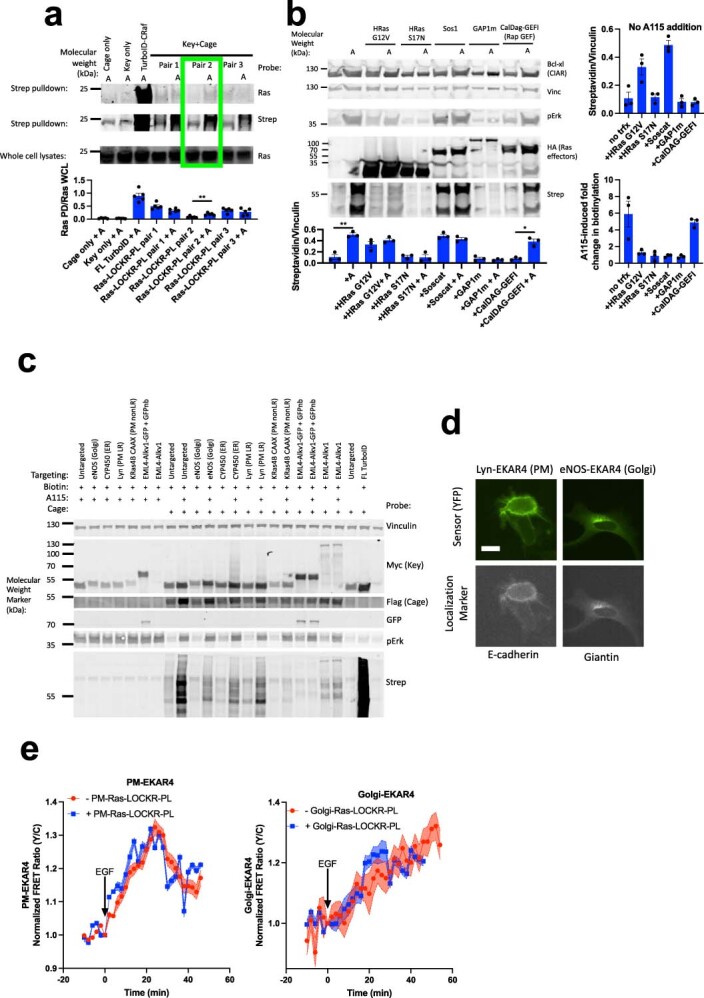
Characterization of Ras-LOCKR-PL. **a–c**, Representative western blots of CIAR-PM-293 cells treated with 500 μM biotin with or without 250 nM A115 (labeled ‘A’) for 16 h. (**a**) Negative controls (Myc-tagged Key or Flag-tagged Cage only), positive control (TurboID-CRaf), and Ras-LOCKR-PL tested designs (Key+Cage) were expressed, and treated with DMSO or with A115. Cell lysates were subjected to a streptavidin pulldown (PD) and the amount of Ras eluted from the streptavidin PD over the amount of Ras from whole cell lysate (WCL) was quantified (n = 5 biologically independent experiments per condition). (**b**) Optimized Ras-LOCKR-PL co-expressed with various HA-tagged Ras mutants/effectors or Rap effector (CalDAG-GEFI) (n = 3 biologically independent experiments per condition). (**c**) Localized Ras-LOCKR-PLs were tested for A115-induced biotinylation in CIAR-PM-293 cells (representative of 3 biologically independent experiments). **d**, Representative epifluorescence images from 3 biologically independent experiments of EKAR4^29^ localized to PM (N-terminus of Lyn)^22^ or Golgi (N-terminus of eNOS)^23^ transfected into 293T cells, which were immunostained for their respective localization markers. **e**, 293T cells transfected with localized EKAR4 and either with or without Ras-LOCKR-PL also localized to same region. Normalized FRET ratio changes over time of these cells stimulated with 100 ng/mL EGF (n = 12 cells per condition). Solid lines indicate representative average timecourse with error bars representing standard error mean (s.e.m.). Bar graphs represent mean ± s.e.m. **p < 0.01, *p < 0.05, unpaired two-tailed Student’s t-test. Scale bar = 10 *μ*m.

The highest dynamic range Ras-LOCKR-PL candidates (boxed in green in Extended Data Fig. 3i) were tested for speed and accuracy at shorter biotinylation times (1 h and 3 h) in A115-treated or EGF-stimulated CIAR-PM-293s (Extended Data Fig. 3j). Of the designs that showed increased biotinylation within 3 h of Ras activation, Ras-LOCKR-PL_Km3C2 (no weakening mutations in latch and three weakening mutations in key) showed consistent Ras-GTP-dependent biotinylation of Ras (Extended Data Fig. 4a); for the remainder of the paper, we refer to this design as Ras-LOCKR-PL. The optimized Key and Cage for Ras-LOCKR-PL required a tighter latch:cage interaction than Ras-LOCKR-S to prevent background signal, which is likely due to the inherent affinity between the two halves of split TurboID (unlike YFP and CFP) and the hours-long labeling time required. To test whether labeling by Ras-LOCKR-PL is dependent on active Ras, we co-expressed it with constitutively active Ras (HRas^G12V^) or with PM-localized catalytic domain of Sos (Sos_cat_) in CIAR-PM-293s. Co-expression of these factors led to increased biotinylation and phospho-Erk levels even without A115 addition. In contrast, co-expression of dominant negative Ras (HRas^S17N^) or the RasGAP Gap1m blunted biotinylation and pErk even with A115 (Extended Data Fig. 4b). Deletion of the RasBD in Ras-LOCKR-PL eliminated A115-induced biotinylation in CIAR-PM-293s (Fig. 3d), demonstrating that Ras-LOCKR-PL relies on Ras-GTP binding to promote labeling. Confirming that labeling by Ras-LOCKR-PL is Ras-GTP specific, co-expression of a RapGEF (CalDAG-GEFI)^9^ did not affect A115-promoted biotinylation (Extended Data Fig. 4b). Together, our data show that Ras-LOCKR-PL is a Ras-GTP-dependent proximity labeler.

We next subcellularly targeted the Key of Ras-LOCKR-PL using the same localization sequences as Ras-LOCKR-S and observed that each construct co-localized with the expected subcellular marker by immunofluorescence (Fig. 3e). Biotinylation by localized Ras-LOCKR-PL in CIAR-PM-293s treated with A115 for 3 h was spatially restricted to expected subcellular regions (Fig. 3f and Extended Data Fig. 4c). Furthermore, we found that the subcellularly localized Ras-LOCKR-PL does not affect global (Extended Data Figs. 3i and 4c) or compartment-specific downstream Erk activity, as measured by subcellularly localized EKAR4 (ref. ^29^) (Extended Data Fig. 4d,e), suggesting that Ras-LOCKR-PL does not substantially buffer signaling downstream of Ras-GTP.

### Identifying signaling effectors inside EML4-Alk granules

**Fig. 4:**
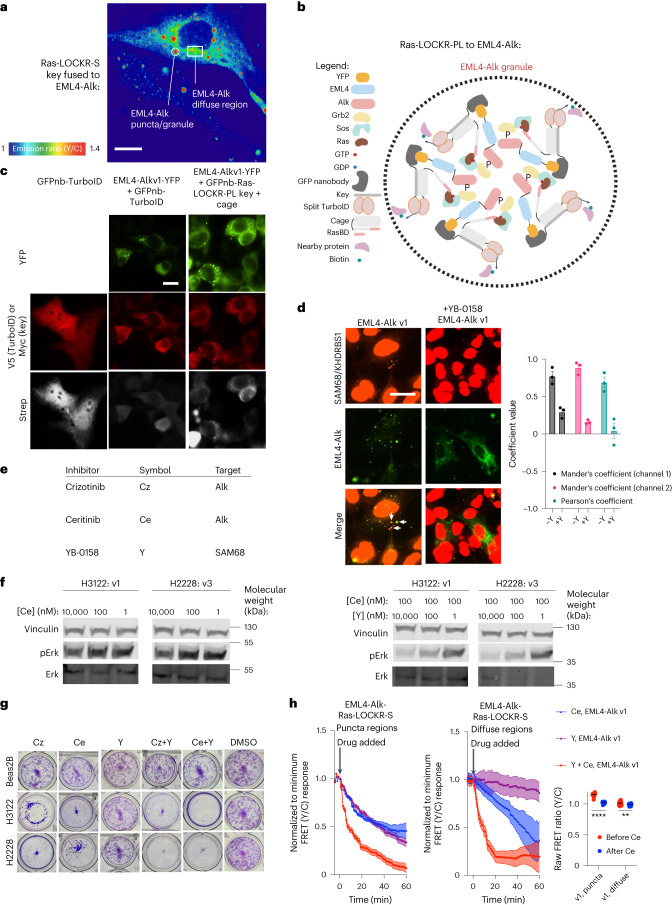
Identifying upstream drivers of oncogenic Ras activity inside EML4-Alk granules. **a**, Representative pseudocolored FRET ratio image of a Beas2B cell transfected with Ras-LOCKR-S localized to EML4-Alk (Key fused to EML4-Alk, Cage untargeted). **b**, Schematic of strategy for localizing Ras-LOCKR-PL to EML4-Alk granules. **c**, Representative epifluorescence images from three biologically independent experiments of Beas2B cells transfected with YFP-fused EML4-Alk variant 1 (v1) and GFP nanobody (GFPnb)-fused V5-tagged TurboID or GFPnb-fused Key of Ras-LOCKR-PL. Cells were treated with 500 μM biotin for 3 h and then probed for biotin labeling. **d**, Left, representative epifluorescence images from three biologically independent experiments of Beas2B cells expressing YFP-tagged EML4-Alk v1, and treated with 1 μM YB-0158 or DMSO for 1 h and immunostained for SAM68. Arrows indicate co-localization of EML4-Alk with SAM68 puncta. Right, co-localization analysis (*n* = 3 biologically independent experiments per condition). **e**, Table of inhibitors used in this figure. **f**, Representative pErk immunoblots from three biologically independent experiments of H3122 (EML4-Alk v1 positive) or H2228 (EML4-Alk v3 positive) cancer patient cells treated with indicated concentrations of inhibitors. **g**, Representative images of crystal violet staining of Beas2B, H3122 and H2228 cells incubated for 1 week with inhibitors to Alk (Cz: 1 μM Crizotinib, Ce: 1 μM Ceritinib), SAM68 (Y: 1 μM YB-0158) or DMSO. **h**, Left, normalized to minimum FRET ratio timecourses of Beas2B cells transfected with Ras-LOCKR-S localized to EML4-Alk v1 and incubated with 10 μM of the inhibitors shown (*n* = 21 cells per condition). Puncta and diffuse regions were analyzed separately (**P = 0.0022 and ****P = 1.6 × 10^−18^). Normalized to minimum FRET ratios are calculated by normalizing the dataset to the condition with the largest decrease in FRET ratios (Ce + Y in both cases), where 0 represents the lowest FRET ratio out of the entire dataset. Right, raw FRET ratios of puncta and diffuse EML4-Alk regions after Alk inhibition for 1 h. Bar graphs represent mean ± s.e.m. ****P < 0.0001 and **P < 0.01, one-way ANOVA. Scale bars, 10 μm.

**Extended Data Fig. 5:**
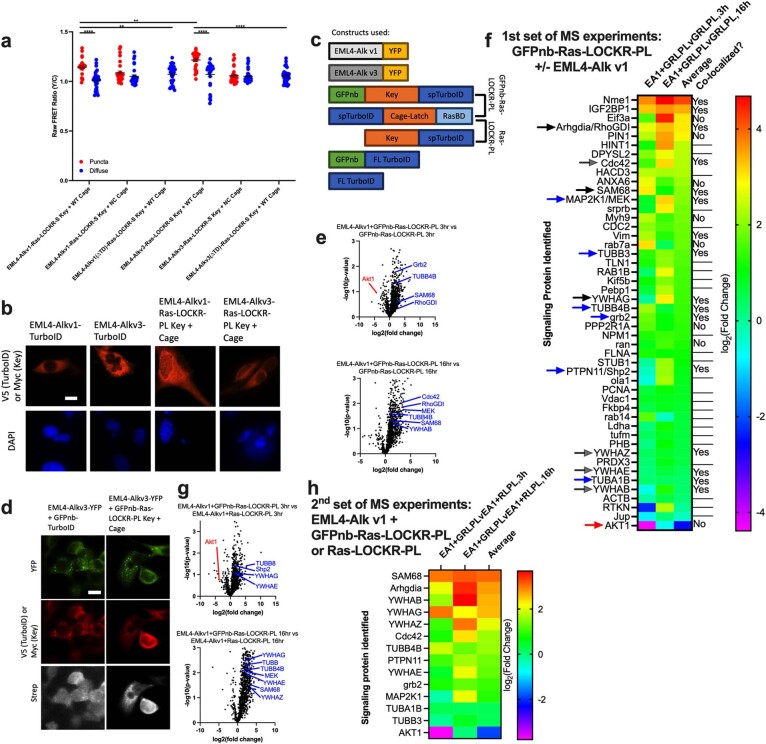
Ras-LOCKR tools identify the Ras activity and environment inside EML4-Alk granules. **a**, Raw FRET ratios of Beas2B cells transfected with Ras-LOCKR-S or NC sensor tethered to EML4-Alk variant 1 (v1)/variant 3 (v3) full length or with trimerization domain deleted (∆TD) (n = 29 cells per condition). Statistics: p-values from left to right: 5.6 × 10^−13^, 0.0045, 0.0082, 6.7 × 10^−12^, 1.3 × 10^−14^. **b**, Representative epifluorescence images from 3 biologically independent experiments of Beas2B cells transfected with V5-tagged TurboID or Myc-tagged Ras-LOCKR-PL tethered to EML4-Alk v1/v3 and immunostained for respective tags. **c**, Domain structures of constructs used. **d**, Representative epifluorescence images from 3 biologically independent experiments of Beas2B (WT lung) transfected with EML4-Alk variant 3 (v3), full length V5-tagged TurboID tethered to GFP nanobody (GFPnb), or Myc-tagged Ras-LOCKR-PL tethered to GFPnb and immunostained for respective tags and biotinylation via fluorescent dye-conjugated streptavidin. **e**, Volcano plot of mass spectrometry results of Beas2B cells expressing either GFPnb-Ras-LOCKR-PL and EML4-Alk-v1 or GFPnb-Ras-LOCKR-PL alone, and incubated for indicated durations with 500 *μ*M biotin. Plotted differences compare enrichment from GFPnb-Ras-LOCKR-PL and EML4-Alk-v1 coexpressing cells against cells expressing GFPnb-Ras-LOCKR-PL alone. P-values derived from two-way student t-test. **f**, From MS data, hits were filtered based on selective labeling within EML4-Alk v1-expressing cells (more than 2-fold change (log_2_(fold change) > 1)), passing a statistical cutoff (p-value cutoff of 0.5 (-log_10_p-value ~ 1.3)) for either the 3 h or 16 h datasets, are signaling proteins as identified in gene ontology analysis, and abundant proteins (proteins related to the proteasome and ribosome) were excluded. Several hits from this dataset were validated by immunostaining in Beas2B cells expressing YFP-tagged EML4-Alk v1. The co-localized column (right-most) indicates whether that hit was co-localized with EML4-Alk v1 granules (yes), not co-localized (no), or not tested (----). For the 14-3-3 proteins (YWHAB/E/G/Z), the co-localization validation is from a 14-3-3 antibody which has an epitope that targets all these 14-3-3 variants. Heat maps (colored by log_2_fold change) display the signaling-related proteins detected in EML4-Alk v1-expressing Beas2B cells. Blue arrows are proteins expected to be enriched in EML4-Alk v1 granules, red arrows are proteins expected to be excluded from EML4-Alk v1 granules, black arrows are new hits that were verified later on to be sequestered in EML4-Alk granules, and gray arrows are known interactors (based on STRING databases) of the validated proteins. **g**, Volcano plot of mass spectrometry results of EML4-Alk v1-expressing Beas2B cells co-expressing either GFPnb-Ras-LOCKR-PL (right) or Ras-LOCKR-PL (left) and treated for indicated durations with 500 *μ*M biotin. P-values derived from two-way student t-test. **h**, Hits identified in first set of MS experiments (**e-f**) were identified in the second set of MS experiments (g), and the log_2_(fold change) from the second set of MS experiments are listed and colored in the heatmap shown. MS data is documented in Supplementary Table 3 (P-values derived from two-way student t-test). Bar graphs represent mean ± s.e.m. ****p < 0.0001, **p < 0.01, ordinary two-way ANOVA. Scale bars = 10 *μ*m.

In addition to membranes, Ras signaling can occur in membrane-less oncogenic granules, such as those formed by EML4-Alk fusions found in non-small cell lung cancers^30^. The most common EML4-Alk fusions, variant 1 (v1) and variant 3 (v3), form cytosolic granules that recruit upstream Ras activators via the Alk kinase domain^5,31^. To measure Ras activity within these structures, we fused the Key of Ras-LOCKR-S to EML4-Alk and co-expressed it in Beas2B lung cells with an untargeted Cage; this did not affect granule formation (Fig. 4a). Consistent with previous reports suggesting increased Ras activity within EML4-Alk-containing granules^5^, we observed increased FRET ratios in punctate regions compared to the diffuse regions of Beas2B cells expressing the EML4-Alk-targeted Ras-LOCKR-S system (Fig. 4a and Extended Data Fig. 5a). In contrast, FRET ratios were low in punctate and diffuse regions in cells expressing Ras-LOCKR-S NC (Extended Data Fig. 5a). Furthermore, when the Key was fused to an EML4-Alk variant that does not form granules (Δ trimerization domain (TD))^5,31^, similarly low levels of FRET were observed (Extended Data Fig. 5a). Thus, Ras-LOCKR-S can measure endogenous Ras activity in membrane-less granules.

Several Ras effectors have been shown to be located within EML4-Alk granules^5^, but an unbiased profiling of the components within this membrane-less Ras signalosome has not been performed due to a lack of appropriate tools. Thus, we generated granule-localized versions of Ras-LOCKR-PL (Fig. 4b). Fusing the Key of Ras-LOCKR-PL to EML4-Alk disrupted granule formation (Extended Data Fig. 5b) but not fusion of the Key to a GFP nanobody (GFPnb) that targets EML4-Alk-YFP (Fig. 4b,c and Extended Data Fig. 5c,d). Ras-LOCKR-PL recruitment to granules resulted in substantial biotinylation inside the granule relative to diffuse regions, consistent with increased Ras signaling inside EML4-Alk granules (Fig. 4c and Extended Data Fig. 5d). In contrast, expressing full-length TurboID (FL TID) fused to a GFPnb in EML4-Alk-YFP-expressing Beas2B cells led to diffusely cytosolic biotinylation (Fig. 4c and Extended Data Fig. 5d), highlighting the ability of Ras-LOCKR-PL to selectively label proteins within Ras signaling microdomains.

To broadly profile the Ras signalosome inside EML4-Alk-containing granules, we performed proteomic experiments comparing labeled proteins in Beas2Bs co-expressing EML4-Alk v1 and GFPnb-Ras-LOCKR-PL versus Beas2Bs expressing GFPnb-Ras-LOCKR-PL alone (Extended Data Fig. 5e,f and Supplementary Table 3). We identified several proteins that were selectively labeled in EML4-Alk v1-expressing cells after 3-h or 16-h biotin incubation. Proteins with increased labeling in EML4-Alk v1-expressing Beas2Bs include previously characterized EML4 interactors (filtering details in legend of Extended Data Fig. 5f), such as tubulin^31^, and known effectors of the Ras/MAPK pathway^5,31^, such as Grb2, MAP2K1/MEK and PTPN11/Shp2. In contrast, Akt, which was shown to be excluded from EML4-Alk granules^5^, had reduced labeling in Beas2B cells expressing EML4-Alk fusions than in those that do not (Extended Data Fig. 5f). These results demonstrate the ability of Ras-LOCKR-PL to identify components of active Ras signalosomes. To determine whether these proteins are indeed enriched in EML4-Alk v1 granules, we performed a second set of proteomic experiments in EML4-Alk v1-expressing Beas2Bs comparing targeted Ras-LOCKR-PL (GFPnb-Ras-LOCKR-PL) to untargeted Ras-LOCKR-PL to identify components selectively enriched in EML4-Alk granules (Extended Data Fig. 5g,h and Supplementary Table 3). All the aforementioned components identified in the first set of mass spectrometry (MS) experiments were also selectively enriched inside EML4-Alk v1 granules in this second set of MS experiments.

**Extended Data Fig. 6:**
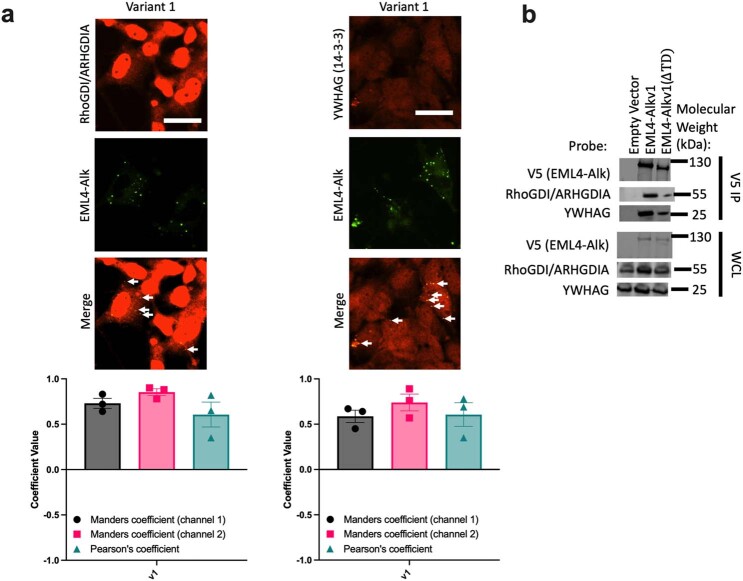
Identification of components sequestered in EML4-Alk granules. **a**, (top) Representative epifluorescence images of Beas2B cells expressing GFP-tagged EML4-Alk v1 and immunostained for hits identified in the mass spectrometry analysis. Arrows indicate co-localization of EML4-Alk v1 with probed protein. (bottom) Colocalization analysis (n = 3 biologically independent experiments per condition). **b**, Representative immunoblot of Beas2B cells transfected with V5-tagged EML4-Alk v1 full length or with trimerization domain deleted (∆TD), subjected to V5 immunoprecipitation, and probed for hits identified in the MS analysis. Bar graphs represent mean ± s.e.m.

We further characterized a subset of the proteins suggested by our proteomic experiments to be enriched in EML4-Alk granules that were not previously known to be present. 14-3-3 proteins (YWHAB/E/G/Z) interact with phosphorylated MAPK pathway components^32^ and were selectively labeled in EML4-Alk v1-expressing Beas2B cells (Extended Data Fig. 5f,h). Immunostaining of Beas2B cells expressing YFP-tagged EML4-Alk v1 confirmed the enrichment of YWHAG in EML4-Alk v1 granules (Extended Data Fig. 6a). YWHAG more robustly co-immunoprecipitated with V5-tagged EML4-Alk v1 than with a granule formation-deficient V5-tagged ΔTD mutant^5,31^ (Extended Data Fig. 6b), suggesting that YWHAG (and likely other 14-3-3 proteins) is enriched in EML4-Alk granules. A regulator of Rho GTPase (RhoGDI)^33,34^ was also selectively labeled in both sets of proteomic experiments (Extended Data Fig. 5e,h) as were proteins that interact with RhoGDI, such as Cdc42 (Extended Data Fig. 5f,h). Immunostaining of YFP-EML4-Alk v1-expressing Beas2Bs confirmed the enrichment of RhoGDI in EML4-Alk v1 granules (Extended Data Fig. 6a), and RhoGDI showed greater co-immunoprecipitation with V5-tagged EML4-Alk v1 than with the V5-tagged EML4-Alk v1 ΔTD mutant (Extended Data Fig. 6b). Thus, Ras-LOCKR-PL can identify components selectively enriched within oncogenic granules.

**Extended Data Fig. 7:**
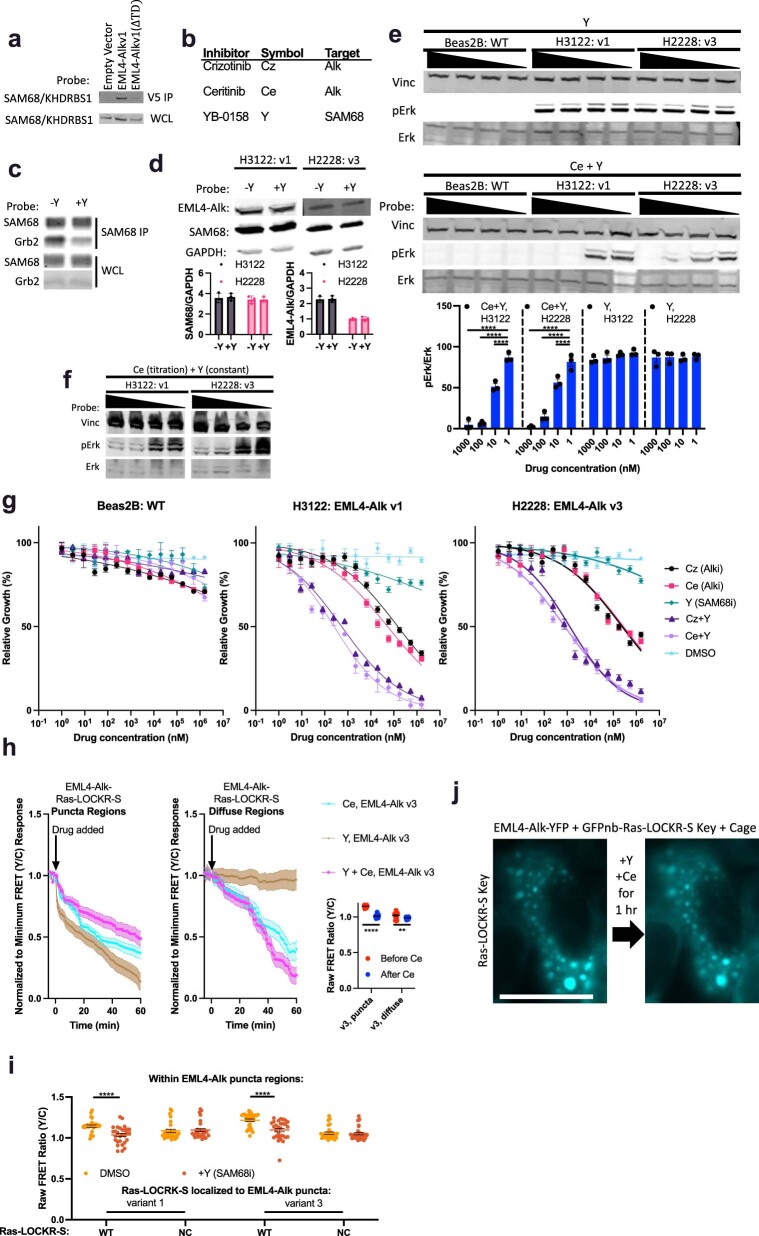
SAM68 regulates Ras signaling inside EML4-Alk granules. **a**, Representative immunoblot from 3 biologically independent experiments of Beas2B cells transfected with V5-tagged EML4-Alk v1 full length or with trimerization domain deleted (∆TD), subjected to V5 immunoprecipitation, and probed for SAM68. The EML4-Alk pulldowns and whole cell lysate levels are the same as Extended Data Fig. 6b. **b**, List of inhibitors used, their abbreviations, and their target. **c**, Representative immunoblot from 3 biologically independent experiments of Beas2B cells treated with 1 *μ*M YB-0158 for 1 h, underwent Sam68 immunoprecipitation for some of the sample, and probed for Sam68 and Grb2. Top: Sam68 immunoprecipitation. Bottom: whole cell lysate. **d**, H3122 or H2228 cells were treated with 1 *μ*M YB-0158 for 24 h and probed for Alk (EML4-Alk) and SAM68. Top: Representative immunoblot. Bottom: densitometry analysis (n = 3 experiments). **e**, (top) Representative immunoblot of Beas2B, H3122, and H2228 cells treated for 1 h with varying concentration of Ceritinib (1nM-1μM) and YB-0158 (1nM-1μM). (bottom) Relative phosphorylated Erk over total Erk quantified (n = 3 experiments per condition). Statistics for **e** from left to right: ****p = 7.3 × 10^−5^, ***p = 0.00072, **p = 0.0043, **p = 0.0047, two-way student t-test. **f**, Representative immunoblot from 3 biologically independent experiments of H3122 and H2228 cells treated for 1 h with either constant concentration of YB-0158 (200 nM) and a range of concentrations of Ceritinib (1nM-1μM) (left) or a constant concentration of Ceritinib (200 nM) and a range of concentrations of YB-0158 (1nM-1μM) (right). **g**, Cell counts of Beas2B, H3122, H2228 cell lines incubated for 1 week with varying concentrations of inhibitors to Alk and/or SAM68 (n = 3 experiments per condition), or DMSO. **h**, (left) Normalized to minimum FRET ratio time-courses of Beas2B cells transfected with Ras-LOCKR-S localized to EML4-Alk v3 and incubated with 1 *μ*M of inhibitors (n = 10 cells per condition). Puncta and diffuse regions were analyzed separately. Normalized to minimum FRET ratios are calculated by normalizing the data set to the condition with the largest decrease in FRET ratios (Ce + Y in both cases) where 0 represents the lowest FRET ratio out of the entire data set. (right) Raw FRET ratios of puncta and diffuse EML4-Alk regions after Alk inhibition for 1 h (n = 21 cells per condition). Statistics: puncta: ****p = 1.5 × 10^−22^, diffuse: **p = 0.0025. **i**, Average raw FRET ratios in the punctate regions of Beas2B cells expressing Ras-LOCKR-S WT or NC sensor fused to EML4-Alk v1. Cells were treated with either DMSO or 1 *μ*M YB-0158 for 1 h (n = 29 cells per condition). Statistics: v1: ****p = 1.7 × 10^−32^, v3: ****p = 6.8 × 10^−13^. **j**, Representative epifluorescence images from 3 biologically independent experiments of Beas2B cells expressing YFP-tagged EML4-Alk v1, GFPnb-Ras-LOCKR-S Key, and Cage. CFP channel is shown indicating localization of Ras-LOCKR-S Key. These cells were incubated with 1 *μ*M YB-0158 and 1 μM Ceritinib for 1 h. Solid lines in **g** indicate IC_50_ fit with points representing average normalized cell count. Solid lines in **h** indicate representative average time with error bars representing standard error mean (s.e.m.). Bar graphs represent mean ± s.e.m. ****p < 0.0001, **p < 0.01 ordinary two-way ANOVA comparing to cells treated with DMSO. Scale bars = 10 *μ*m.

SAM68/KHDRBS1 showed increased labeling within EML4-Alk v1 granules in both of our comparative proteomic analyses (Extended Data Fig. 5e–h). As SAM68 interacts with the Ras effector Grb2 via its SH3 domains^35^ (also enriched in EML4-Alk-expressing Beas2B cells (Extended Data Fig. 5e–h)) and is associated with several cancer types^36^, we explored the function of SAM68 in EML4-Alk granules. Consistent with the increased labeling, immunostaining showed that the cytosolic pool of SAM68 was enriched in EML4-Alk v1 granules of YFP-tagged EML4-Alk v1-expressing Beas2Bs (Fig. 4d; SAM68 is an RNA-binding protein and is mostly localized in the nucleus^36^). SAM68 also co-immunoprecipitated with V5-tagged EML4-Alk v1 but not with the V5-tagged EML4-Alk v1 ΔTD mutant^5,31^ (Extended Data Fig. 7a), suggesting that SAM68 is indeed sequestered to EML4-Alk granules.

We next explored if SAM68 has functional roles in EML4-Alk signaling and proliferation using the small molecule YB-0158 (refs. ^37,38^) (Y) (Fig. 4e). YB-0158 has been reported to bind to SAM68 (ref. ^37^) and directly disrupt its interaction with SH3 domain-containing proteins, such as Src^37^. We observed that treating cells with YB-0158 for 1 h blocked Grb2 co-immunoprecipitation with SAM68 (Extended Data Fig. 7b–d). We used YB-0158 to probe SAM68’s contribution to signaling in the patient-derived EML4-Alk cancer cell lines H3122 (EML4-Alk v1) and H2228 (EML4-Alk v3). One-hour incubation of YB-0158 minimally diminished pErk levels in these cells (Fig. 4f and Extended Data Fig. 7e). As in previous studies^31^, Alk inhibitor (Ceritinib) treatment for 1 h led to only a slight decrease in pErk levels (Fig. 4f). However within 1 h in H3122 and H2228 cells, YB-0158 dose dependently decreased pErk levels in the presence of a fixed concentration of Ceritinib (Fig. 4f), and a fixed concentration of YB-0158 facilitated inhibition of pErk by Ceritinib (Extended Data Fig. 7f). In H3122 and H2228 cells, 1-h co-incubation with Ceritinib and YB-0158 decreased pErk levels more than either inhibitor alone (Extended Data Fig. 7e). YB-0158 (~20% at highest concentration) and Ceritinib (~70%) partially inhibited, whereas co-incubation completely inhibited (~95%), cell growth of EML4-Alk-expressing cancer cells (Extended Data Fig. 7g). Co-treatment with YB-0158 and Ceritinib decreased colony formation (Fig. 4g) more than single-drug treatment, and this effect was specific to EML4-Alk-expressing cancer cells. Our results with YB-0158 suggest that SAM68 contributes to EML4-Alk-mediated oncogenic signaling and cell growth, but YB-0158’s polypharmacology must be kept in mind (‘Discussion’).

We probed the subcellular Ras activity effects of YB-0158 using EML4-Alk-targeted Ras-LOCKR-S (Fig. 4a) to gain more mechanistic insight into how disrupting SAM68:Grb2 interactions affects oncogenic MAPK signaling in EML4-Alk-expressing cells. Within 1 h, YB-0158 decreased SAM68 enrichment in EML4-Alk granules but did not dissolve the granules themselves (Fig. 4d), demonstrating that SAM68 is not involved in granule formation. Timecourse imaging in Beas2B cells expressing EML4-Alk-targeted Ras-LOCKR-S showed that 1-h treatment with Ceritinib alone decreased Ras activity approximately 50% (calculation details in figure legends; Fig. 4h and Extended Data Fig. 7h) inside EML4-Alk v1 and v3 granules but did not change granule size or number. As EML4-Alk has both a punctate and diffuse pool, we investigated whether Ceritinib also decreased Ras activity outside the granule (diffuse regions) (Fig. 4a). Although the diffuse regions had lower basal levels of Ras activity (Fig. 4h and Extended Data Fig. 7h, right), 1-h Ceritinib treatment led to an approximately 60% decrease (Fig. 4h and Extended Data Fig. 7h) in Ras activity in the diffuse regions, suggesting that EML4-Alk in the diffuse regions still signals to Ras. In contrast, 1-h YB-0158 treatment decreased Ras activity primarily within the granule (~65%) but not in the diffuse regions (~5%) (Fig. 4h and Extended Data Fig. 7h,i), suggesting that SAM68 enhances Ras activity exclusively within the granules. One-hour co-incubation of Ceritinib and YB-0158 led to the most marked decrease in Ras activities in both the punctate (~90%) and diffuse (~80%) regions (Fig. 4h and Extended Data Fig. 7h) but did not disrupt EML4-Alk granules (Extended Data Fig. 7j), demonstrating the utility of this combination drug regimen in ensuring blockage of oncogenic Ras activity. Altogether, these acute timecourse imaging experiments demonstrate that YB-0158 can modulate Ras signaling specifically within EML4-Alk granules, presumably through SAM68.

## Discussion

Design of genetically encoded biosensors that match sensor sensitivity to the biologically relevant concentration range of targets is challenging due to the limitations of current engineering methods. Here we show that computational design methodologies together with the LOCKR switch platform enable the development of sensors of endogenous Ras-GTP, which has eluded previous biosensor design efforts (Fig. 1a). Our Ras-GTP sensors allow the real-time measurement of subcellular endogenous Ras activity (Ras-LOCKR-S) at the single-cell level and the profiling of Ras-GTP interactomes using proximity labeling (Ras-LOCKR-PL)^39–41^

Our sensors can measure endogenous Ras activity not only at the PM and endomembranes (Golgi) but also in membrane-less, cytosolic granules. Targeting Ras-LOCKR to EML4-Alk-containing granules led to identification of several unanticipated factors inside these granules, including SAM68, a potential upstream modulator of aberrant Ras activity. A compound that modulates SAM68, YB-0158 (Extended Data Fig. 7c), reduces SAM68 sequestration into EML4-Alk granules (Fig. 4d); SAM68 sequestration into EML4-Alk granules may involve SAM68’s proline-rich motifs^35^ binding to the two SH3 domains of Grb2 (ref. ^42^). The SAM68:Grb2 interaction may prolong Grb2 residency time within granules to activate Sos^43^ and, thus, drive Ras activation. Although our finding that YB-0158 treatment was able to rapidly decrease Ras-GTP levels in granules selectively over diffuse regions (Fig. 4h) in EML4-Alk-expressing cells is consistent with this hypothesis, more detailed biochemical analyses are needed to confirm the functional role of SAM68 in granules due to YB-0158’s known polypharmacology^37,38^. YB-0158 affects the Wnt/β-catenin and NF-κB transcriptional programs and the gene expression of several cell cycle markers, proliferation inhibitors and cancer markers^37,38^, but the short timescales (1 h) in which we see effects on Ras activation and downstream Erk signaling (Fig. 4h and Extended Data Fig. 7c,d) support the notion that YB-0158 acts by disrupting SAM68’s localization within EML4-Alk granules.

The Ras sensors described here should be useful for profiling Ras activities in Ras-driven cancers, their responses to drugs and mechanisms of drug resistance^44,45^. However, the sensors also have limitations. Unavoidable in any biosensor application, the Ras-LOCKR tools contain a target-binding domain (here, RasBD) that could potentially buffer Ras-GTP and alter downstream signaling. Although Ras-LOCKR-PL expression does not affect localized downstream signaling (Extended Data Fig. 4e), there are other signaling pathways modulated by Ras-GTP that could be altered.

Our results illustrate the power of computational design methodology to generate sensors for intracellular mapping of the activities, mechanisms and functions of physiologically relevant molecules, such as activated Ras. We envision that the tools and methods described here can be used to design a wide range of sensors for other targets, accelerating biological and translational discovery.

## Methods

### Computational grafting of sensing domains onto latch domain

The first 7–11 amino acids from the RasBD of CRaf were grafted using RosettaScripts GraftSwitchMover into all α-helical registers between residues 610 and 644 of the latch domain within the Cage protein. The resulting Cages were energy minimized using Rosetta fast relax and visually inspected, and typically fewer than 10 designs were selected for subsequent cellular characterization. See Supplementary Table 4 for details of the software.

### Plasmid construction

All plasmids constructed here are using the pcDNA 3.1 backbone (unless otherwise indicated) and were produced by GenScript. Some plasmids were gifts from collaborators or ordered from Addgene. See Supplementary Table 4 for details of the reagents.

### Cell culture and transfection

HEK293T, HEK293, HEK293F, HEK293-FlpIn TRex, HeLa and Ras-less MEF cell lines were cultured in DMEM containing 1 g L^−1^ glucose and supplemented with 10% (v/v) FBS and 1% (v/v) penicillin–streptomycin (Pen–Strep). Beas2B, Jurkat, H3122 and H2228 cells were cultured in RPMI 1640 with 10% (v/v) FBS and 1% Pen–Strep. All cells were grown in a humidified incubator at 5% CO_2_ and 37 °C.

Before transfection, all cells were plated onto sterile poly-d-lysine-coated plates or dishes and grown to 50–70% confluence. HEK293T, HEK293 and Beas2B cells were transfected using TurboFectin 8; HEK293F cells were transfected with PEI-MAX; Jurkat cells were transfected with Lipofectamine LTX; and all other cells/conditions were transfected with FuGENE HD. For EML4-Alk transfections, cells were grown an additional 48 h before imaging to allow puncta formation. For all other transfections, cells were grown for an additional 16–24 h before imaging. All cells underwent serum starvation for 16 h unless indicated. See Supplementary Table 4 for details of the reagents.

### General procedures for bacterial protein production and purification

Except for purification of RBD, the *Escherichia coli* Lemo21(DE3) strain was transformed with a pET29b^+^ plasmid encoding the synthesized gene of interest. Cells were grown for 24 h in liquid broth medium supplemented with kanamycin. Cells were inoculated at a 1:50 ml ratio in Studier TBM-5052 autoinduction medium supplemented with kanamycin, grown at 37 °C for 2–4 h and then grown at 18 °C for an additional 18 h. Cells were collected by centrifugation at 4,000*g* at 4 °C for 15 min and resuspended in 30 ml of lysis buffer (20 mM Tris-HCl, pH 8.0, 300 mM NaCl, 30 mM imidazole, 1 mM PMSF and 0.02 mg ml^−1^ DNase). Cell resuspensions were lysed by sonication for 2.5 min (5-s cycles). Lysates were clarified by centrifugation at 24,000*g* at 4 °C for 20 min and passed through 2-ml Ni-NTA nickel resin pre-equilibrated with wash buffer (20 mM Tris-HCl, pH 8.0, 300 mM NaCl and 30 mM imidazole). The resin was washed twice with 10 column volumes (Cversus) of wash buffer and then eluted with 3 Cversus elution buffer (20 mM Tris-HCl, pH 8.0, 300 mM NaCl and 300 mM imidazole). The eluted proteins were concentrated using Ultra-15 Centrifugal Filter Units and further purified by using a Superdex 75 Increase 10/300 GL size exclusion column in TBS (25 mM Tris-HCl, pH 8.0, and 150 mM NaCl). Fractions containing monomeric protein were pooled, concentrated and snap frozen in liquid nitrogen and stored at −80 °C. See Supplementary Table 4 for details of the reagents.

### Procedure to purify RBD from mammalian cells

RBD proteins were produced in HEK293F cells grown in suspension using HEK293F expression medium at 33 °C, 70% humidity and 8% CO_2_, rotating at 100*g*. The cultures were transfected using PEI-MAX with cells grown to a density of 3 × 10^6^ cells per milliliter and cultivated for 3 d. Supernatants were clarified by centrifugation (5 min at 4,000*g*) and addition of polydiallyldimethylammonium chloride solution to a final concentration of 0.0375% and a second spin (5 min at 4,000*g*).

His-tagged RBD was purified from clarified supernatants via a batch bind method, where each clarified supernatant was supplemented with 1 M Tris-HCl, pH 8.0, to a final concentration of 45 mM and 5 M NaCl to a final concentration of 310 mM. Talon cobalt affinity resin was added to the treated supernatants and allowed to incubate for 15 min with gentle shaking. Resin was collected using vacuum filtration with a 0.2-mm filter and transferred to a gravity column. The resin was washed with 20 mM Tris, pH 8.0, and 300 mM NaCl, and the protein was eluted with 3 Cversus of 20 mM Tris, pH 8.0, 300 mM NaCl and 300 mM imidazole. The batch bind process was then repeated, and the first and second elutions were combined. SDS-PAGE was used to assess purity. After immobilized metal affinity chromatography purification, the elution was concentrated and applied to a Cytiva S200 Increase column equilibrated with 20 mM Tris and 150 mM NaCl, pH 8.0, and the peak of interest was collected and quantified using A280. See Supplementary Table 4 for details of the reagents.

### Cell counting to measure cell proliferation

Jurkat, Beas2B, H3122 and H2228 cell lines were seeded in six-well plates at 10,000 cells per well. Cell numbers were quantified using a hemacytometer each day for 7 d.

### Colony formation assay

Beas2B, H3122 and H2228 cell lines were seeded in 24-well plates at 100 cells per well. After 1–2 weeks to allow cell growth, cells were washed once with PBS, fixed with 4% paraformaldehyde (PFA) in PBS for 10 min, stained with 2.5 mg ml^−1^ crystal violet stain dissolved in 20% methanol for 10 min and then washed six times with PBS. Images were captured using a ZOE Fluorescent Cell Imager (Bio-Rad).

### Immunostaining

293T, HeLa, Jurkat and Beas2B cell lines were seeded onto 24-well glass-bottom plates. After transfection and drug addition, cells were fixed with 4% PFA in 2× PHEM buffer (60 mM PIPES, 50 mM HEPES, 20 mM EGTA, 4 mM MgCl_2_, 0.25 M sucrose, pH 7.3) for 10 min at room temperature, permeabilized with 100% methanol for 10 min at 4 °C, washed with PBS three times at room temperature, blocked in 1% BSA in PBS for 30 min at room temeprature, incubated with primary antibody overnight at 4 °C, washed with PBS three times and incubated with DAPI, neutravidin-DyLight 650 and/or secondary antibody for 1 h at room temperature with an aluminum foil cover. Cells were then washed with PBS three times and mounted for epifluorescence imaging. All images were analyzed in ImageJ. See Supplementary Table 4 for details of the reagents.

### Immunoblotting and immunoprecipitation

Cells expressing indicated constructs and incubated with indicated drugs were plated, transfected and labeled as described in the figure legends. Cells were then transferred to ice and washed two times with ice-cold DPBS. Cells were then detached from the well by addition of 1× RIPA lysis buffer (50 mM Tris, pH 8, 150 mM NaCl, 0.1% SDS, 0.5% sodium deoxycholate, 1% Triton X-100, 1× protease inhibitor cocktail, 1 mM PMSF, 1 mM Na_3_VO_4_, 1% NP-40) and either scraping of cells or rotation on a shaker for 30 min at 4 °C. Cells were then collected and vortexed for at least 5 s every 10 min for 20 min at 4 °C. Cells were then collected and clarified by centrifugation at 13,000*g* for 10 min at 4 °C. The supernatant was collected and underwent Pierce BCA assay to quantify total protein amounts.

For immunoblotting, whole cell lysate protein amounts were normalized across samples in the same gel, mixed with 4× loading buffer before loading, incubated at 95 °C for 5 min and then 4 °C for 5 min and separated on Any kDa SDS-PAGE gels. Proteins separated on SDS-PAGE gels were transferred to nitrocellulose membranes via a TransBlot system (Bio-Rad). The blots were then blocked in 5% milk (w/v) in Tris-buffered saline with 0.1% Tween 20 (TBST) for 1 h at room temperature. Blots were washed with TBST three times and then incubated with indicated primary antibodies in 1% milk (w/v) in TBST overnight at 4 °C. Blots were then washed with TBST three times and incubated with LI-COR dye-conjugated secondary antibodies (LI-COR 680/800 or streptavidin-LI-COR 800) in 1% milk (w/v) in TBST for 1 h at room temperature. The blots were washed with TBST three times and imaged on an Odyssey IR imager (LI-COR). Quantitation of western blots was performed using ImageJ on raw images.

For immunoprecipitation, agarose beads were either pre-loaded with streptavidin (high-capacity streptavidin beads) or loaded by 3× lysis buffer washes and then addition of 1 mg ml^−1^ indicated antibodies at 4 °C on an orbital shaker for 3 h. Beads were then washed two times in lysis buffer. Whole cell lysate protein amounts were normalized across samples, and protein samples were added to beads (at least 100 μg per sample) either at room temperature for 1 h for streptavidin beads or at 4 °C on an orbital shaker overnight. Beads were then washed two times in lysis buffer and one time in TBS and then mixed with 4× loading buffer sometimes containing 2 mM biotin and 20 mM DTT^46^ for streptavidin pulldowns. The remaining portion of the protocol is the same as immunoblotting. See Supplementary Table 4 for details of the reagents, including antibody dilutions. All uncropped immunoblots are shown in Supplementary Fig. 1.

### MS analysis

Cells expressing indicated constructs and incubated with indicated drugs were plated, transfected and labeled as described in the figure legends. Cells were then transferred to ice and washed two times with ice-cold DPBS, detached from the well by addition of 1× RIPA lysis buffer (50 mM Tris, pH 8, 150 mM NaCl, 0.1% SDS, 0.5% sodium deoxycholate, 1% Triton X-100, 1× protease inhibitor cocktail, 1 mM PMSF, 1 mM Na_3_VO_4_, 1% NP-40) and scraping of cells, collected and vortexed for at least 5 s every 10 min for 20 min at 4 °C and collected and clarified by centrifugation at 20,000*g* for 10 min at 4 °C. The supernatant was collected and underwent Pierce BCA assay to quantify total protein amounts.

Next, 50 μl of high-capacity streptavidin agarose beads was washed two times in lysis buffer. Whole cell lysate protein amounts were normalized across samples, and protein samples were added to beads (at least 100 μg per sample) at room temperature for 1 h. Beads were then washed two times with lysis buffer, one time with 1 M KCl, one time with 0.1 M Na_2_CO_3_, two times with 2 M urea and two times with TBS. Beads were re-suspended in 50 μl of denaturing buffer (6 M guanidinium chloride, 50 mM Tris containing 5 mM TCEP and 10 mM CAM with TCEP and CAM added fresh every time), inverted a few times and heated to 95 °C for 5 min. The bead slurry was diluted with 50 μl of 100 mM TEAB, and 0.8 μg of LysC was added per sample with the pH adjusted to 8–9 using 1 M NaOH. This mixture was agitated on a thermomixer at 37 °C for 2 h at 1,400 r.p.m. Afterwards, samples were diluted two times with 100 μl of 100 mM TEAB with 0.8 μg of sequencing-grade trypsin per sample, with the pH adjusted to 8–9 using 1 M NaOH. This mixture was agitated on a thermomixer at 37 °C for 12–14 h at 800 r.p.m. After overnight trypsinization, samples were diluted two times with 200 μl of Buffer A (5% acetonitrile with 0.1% TFA) containing 1% formic acid. These samples were inverted a few times and pH adjusted to 2–3 using 100% formic acid. StageTips for peptide desalting were prepared by extracting out plugs from C18 matrices, shoved down a 200-μl tip and pressed with a plunger for flatness. Using these StageTips, 50 μl of Buffer B (80% acetonitrile with 0.1% TFA) was passed through at 4,000*g* for 1 min, followed by 5 μl of Buffer A at 4,000*g* for 1 min. The supernatant of the samples was added to StageTips and spun down at 4,000*g* for 5 min. Then, 50 μl of Buffer A was added and spun down at 4,000*g* for 2.5 min. Then, 50 μl of Buffer B was added to StageTips, and a syringe pump was applied to elute samples.

Peptide samples were separated on an EASY-nLC 1200 System (Thermo Fisher Scientific) using 20-cm-long fused silica capillary columns (100-µm ID, laser pulled in-house with a Sutter Instrument P-2000) packed with 3-μm, 120-Å reversed-phase C18 beads (Dr. Maisch). The LC gradient was 90 min long with 5−35% B at 300 nl min^−1^. LC solvent A was 0.1% (v/v) aqueous acetic acid, and LC solvent B was 20% 0.1% (v/v) acetic acid and 80% acetonitrile. MS data were collected with a Thermo Fisher Scientific Orbitrap Fusion Lumos using a data-dependent data acquisition method with an Orbitrap MS1 survey scan (R = 60,000) and as many Orbitrap HCD MS2 scans (R = 30,000) as possible within the 2-s cycle time.

### Computation of MS raw files

Data.raw files were analyzed by MaxQuant/Andromeda version 1.5.2.8 using protein, peptide and site false discovery rates (FDRs) of 0.01 and a score minimum of 40 for modified peptides and 0 for unmodified peptides; delta score minimum of 17 for modified peptides and 0 for unmodified peptides. Tandem mass spectrometry (MS/MS) spectra were searched against the UniProt human database (updated 22 July 2015). MaxQuant search parameters were as follows: Variable modifications included Oxidation (M) and Phospho (S/T/Y). Carbamidomethyl (C) was a fixed modification. Maximum missed cleavages was 2, enzyme was Trypsin/P and maximum charge was 7. The MaxQuant ‘match between runs’ feature was enabled. The initial search tolerance for FTMS scans was 20 ppm and 0.5 Da for ITMS MS/MS scans.

### MaxQuant output data processing

MaxQuant output files were processed, statistically analyzed and clustered using the Perseus software package version 1.5.6.0. Human Gene Ontology (GO) terms (GOBP, GOCC and GOMF) were loaded from the ‘mainAnnot.homo_sapiens.txt’ file downloaded on 3 February 2020. Expression columns (protein and phosphopeptide intensities) were log_2_ transformed and normalized by subtracting the median log_2_ expression value from each expression value of the corresponding data column. Potential contaminants, reverse hits and proteins identified only by site (biotinylation) were removed. Reproducibility between liquid chromatography with tandem mass spectrometry (LC–MS/MS) experiments was analyzed by column correlation (Pearson’s *r*), and replicates with a variation of *r* > 0.25 compared to the mean *r* values of all replicates of the same experiment (cell line or knockdown experiment) were considered outliers and excluded from the analyses. Data imputation was performed in Perseus using a modeled distribution of MS intensity values downshifted by 1.8 and having a width of 0.2. Hits were further filtered using GO analysis (signaling pathways) via the PANTHER database. See Supplementary Table 4 for details of the reagents.

### In vitro fluorescence characterization

A Synergy Neo2 Microplate Reader (BioTek) was used for all in vitro fluorescence measurements. Assays were performed in 1× PBS. The purified protein components (+50 μM DFHBI-1T for mFAP2a experiments) were placed in 96-well, black-well, clear-bottom plates, centrifuged at 1,000*g* for 1 min and incubated for 30 min at room temperature to enable pre-equilibration. Fluorescence measurements in the absence of target were taken every 1 min after injection (0.1-s integration and 10-s shaking during intervals) at the indicated wavelengths. For FRET spectra, the wells were excited at wavelengths indicated in the figure legends, and the respective FRET was recorded at 5-nm intervals. See Supplementary Table 4 for details of the reagents.

### Time-lapse epifluorescence imaging

Cells were washed twice with FluoroBrite DMEM imaging media and subsequently imaged in the same media in the dark at room temperature. Forskolin, EGF, A115 and *α*-CD3*ε* + *α*-CD28 were added as indicated. LFA-1 stimulation by ICAM-1 was done by coating plates as described in a previous report^47^. Epifluorescence imaging was performed on a Yokogawa CSU-X1 spinning disk confocal microscope with either a Lumencor Celesta light engine with seven laser lines (408, 445, 473, 518, 545, 635 and 750 nm) or a Nikon LUN-F XL laser launch with four solid-state lasers (405, 488, 561 and 640 nm), ×40/0.95 NA objective or ×60/1.4 NA oil immersion objective and a Hamamatsu ORCA-Fusion scientific CMOS camera, both controlled by NIS Elements 5.30 software (Nikon). The following excitation/FRET filter combinations (center/bandwidth in nm) were used: CFP: EX445 EM483/32, CFP/YFP FRET: EX445 EM542/27, YFP: EX473 EM544/24, GFP: EX473 EM525/36, RFP: EX545 EM605/52, Far Red (for example, Alexa Fluor 647): EX635 EM705/72. Exposure times were 100 ms for acceptor direct channel and 500 ms for all other channels, with no EM gain set and no ND filter added. Cells that were too bright (acceptor channel intensity is 3 s.d. above mean intensity of transfected cells, which is quantified across multiple experiments) or with substantial photobleaching before drug addition were excluded from analysis. All epifluorescence experiments were subsequently analyzed using ImageJ. Bright-field images were acquired on the ZOE Fluorescent Cell Imager (Bio-Rad). See Supplementary Table 4 for details of the reagents.

### FRET biosensor analysis

Raw fluorescence images were corrected by subtracting the background fluorescence intensity of a cell-free region from the fluorescence intensities of biosensor-expressing cells. Cyan/yellow FRET ratios were then calculated at each timepoint (*R*), which represents raw FRET ratios. For some curves, the resulting timecourses were normalized by dividing the FRET ratio at each timepoint by the basal ratio value at time zero (*R*/*R*_0_), which was defined as the FRET ratio at the timepoint immediately preceding drug addition (*R*_0_)^48^. Graphs were plotted using GraphPad Prism 8 (GraphPad Software). Signal-to-noise ratio analysis was done on single-cell timecourses by taking the ratio of the maximum FRET ratio change to the standard deviation of the baseline before drug addition^49^.

For generating pseudocolored FRET ratio images, the background was eliminated to enhance image quality. To do so, the average background signal was calculated on ImageJ, and each channel was background subtracted. Afterwards, the FRET channel images were divided by the CFP channel images and were colored by the physics lookup table.

### Co-localization analysis

For co-localization analysis, cell images were individually thresholded and underwent Coloc 2 analysis on ImageJ. For targets that have substantial fluorescence in the nucleus (for example, SAM68), a zoomed-in region that excludes the nucleus was used for co-localization analysis. Mander’s coefficient, which ranges from 0 to 1, with 1 being 100% co-localized, measures the spatial overlap of one imaging channel (for example, EML4-Alk-YFP) with another imaging channel (for example, immunostained SAM68). Pearson’s coefficient compares the pixel intensity of one channel with another channel. Pearson’s coefficient values can range from −1 to 1, with −1 meaning inversely proportional and 1 meaning same pixel intensities.

### Quantification of cellular puncta

For analysis of puncta number, cell images were individually thresholded and underwent particle analysis with circularity (>0.5) and size cutoffs (>1 μm^2^) in ImageJ.

### Graphics

All schematics were generated using BioRender.

### Statistics and reproducibility

No statistical methods were used to predetermine the sample size. No sample was excluded from data analysis, and no blinding was used. All data were assessed for normality. For normally distributed data, pairwise comparisons were performed using unpaired two-tailed Student’s *t*-tests, with Welch’s correction for unequal variances used as indicated. Comparisons between three or more groups were performed using ordinary one-way or two-way ANOVA as indicated. For data that were not normally distributed, pairwise comparisons were performed using the Mann–Whitney *U*-test, and comparisons between multiple groups were performed using the Kruskal–Wallis test. All data shown are reported as mean ± s.e.m., and error bars in figures represent s.e.m. of biological triplicates. All data were analyzed and plotted using GraphPad Prism 8, including nonlinear regression fitting.

### Reporting summary

Further information on research design is available in the Nature Portfolio Reporting Summary linked to this article.

## Online content

Any methods, additional references, Nature Portfolio reporting summaries, source data, extended data, supplementary information, acknowledgements, peer review information; details of author contributions and competing interests; and statements of data and code availability are available at 10.1038/s41587-023-02107-w.

## Supplementary information

Supplementary Information Supplementary Fig. 1 (uncropped gel blots).

Reporting Summary

Supplementary Tables 1–4 Supplementary Table 1: LOCKR-S candidates tested. RBD-LOCKR-S in Tab 1 or Ras-LOCKR-S in Tab 2 that were tested in this study. Domain structure, sequence and performance to either RBD expression (RBD-LOCKR-S) or EGF stimulation (Ras-LOCKR-S) are documented. Supplementary Table 2: LOCKR-PL candidates tested. RBD-LOCKR-PL in Tab 1 or Ras-LOCKR-PL in Tab 2 that were tested in this study. Domain structure, sequence and performance to either RBD expression (RBD-LOCKR-PL) or EGF stimulation (Ras-LOCKR-PL) are documented. Supplementary Table 3: MS data of Ras-active EML4-Alk granules. Details of MS experiments are in the figure legend of Extended Data Fig. 5. Supplementary Table 4: Resources and identifiers. Details of cells, molecules and software used in this study.

## Data Availability

The data that support the findings of this study are available from figshare (https://figshare.com/projects/Computationally_designed_sensors_detect_Ras_activity_and_signaling_effectors_at_subcellular_resolution/186933)^50^. Proteomic raw data are available on PRIDE (https://massive.ucsd.edu/ProteoSAFe/dataset.jsp?task=66ee1bb3919049f0a7465bb199e2e324)^51^. Sequences of Ras-LOCKR-S and Ras-LOCKR-PL are in Supplementary Tables 1 and 2.

## Code availability

The code and design models used in this study are available from Zenodo (https://zenodo.org/records/10202412?token=eyJhbGciOiJIUzUxMiJ9.eyJpZCI6IjZhY2ZkM2E2LTcyMDQtNDNjMS05NDRmLTczZTNjODk0MjJiNiIsImRhdGEiOnt9LCJyYW5kb20iOiI1NjhhMWQxMzZiMmEwNTQwODIxMjdiYjBjM2FmOWE1YSJ9.D7retFS99L6nbkRCmS4Oojj109D83doIKhkMYVzMNyPqmpVZwQnErwrCiG8YfBQ-Vjh_vjyOGXw6OPgk1VsIsw)^52^.
