# Supplementary material for: Computationally designed sensors detect endogenous Ras activity and signaling effectors at subcellular resolution
Source: Nat Biotechnol. 2024 Jan 25;42(12):1888–98. doi: 10.1038/s41587-023-02107-w (PMC11631767; doi:10.1038/s41587-023-02107-w)

nature biotechnology

Article https://doi.org/10.1038/s41587-023-02107-w

Computationally designed sensors detect endogenous Ras activity and signaling effectors at subcellular resolution

In the format provided by the authors and unedited

Figure 3d:

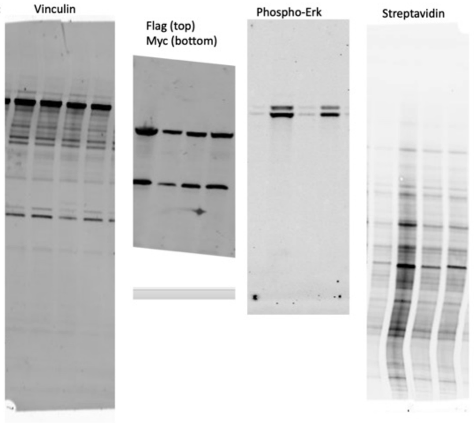

Extended Data Figure 3b (14AA linker)

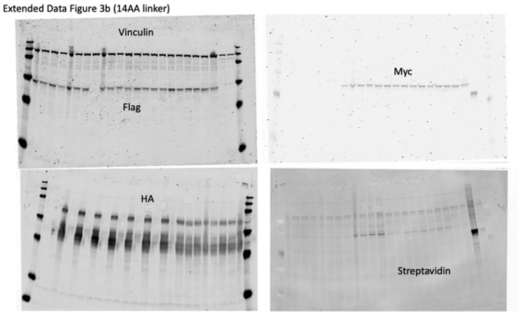

Extended Data Figure 3b (28AA linker)

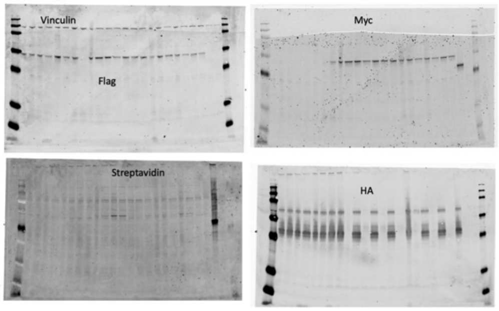

Extended Data Figure 3d

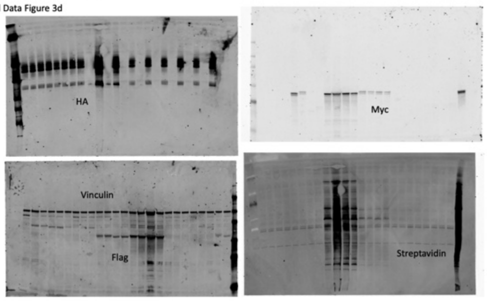

Extended Data Figure 3g

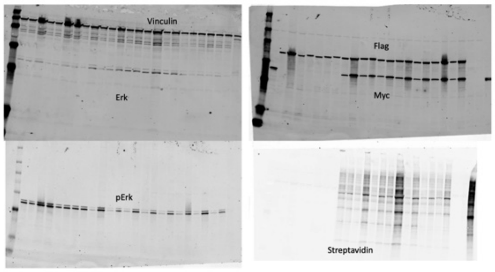

Extended Data Figure 3h

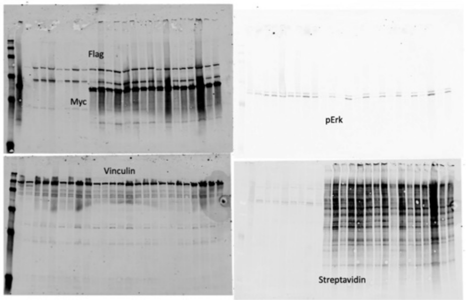

Extended Data Figure 3i Key 1

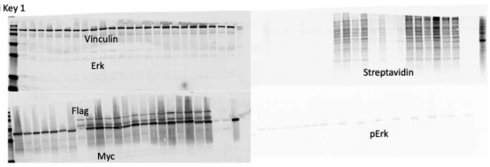

Extended Data Figure 3i Key 2

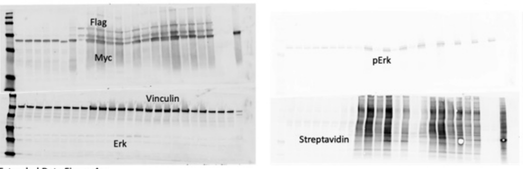

Extended Data Figure 3j 1 hour

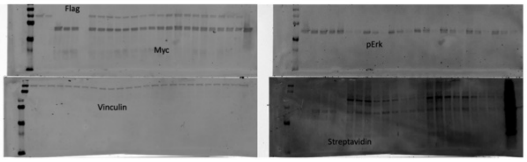

Extended Data Figure 3j 3 hour

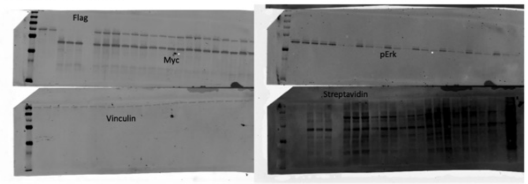

Extended Data Figure 4a

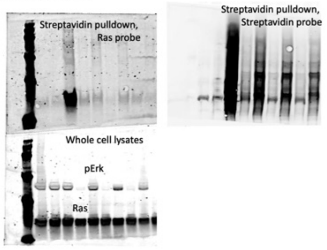

Extended Data Figure 4b

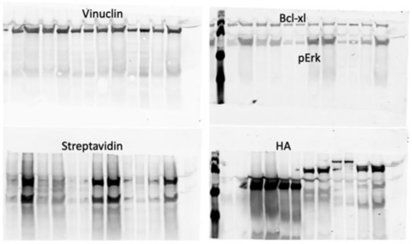

Extended Data Figure 4c

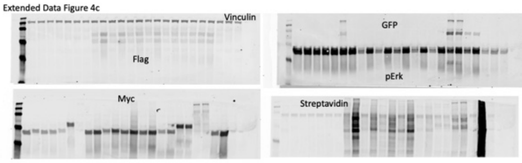

Figure 4f

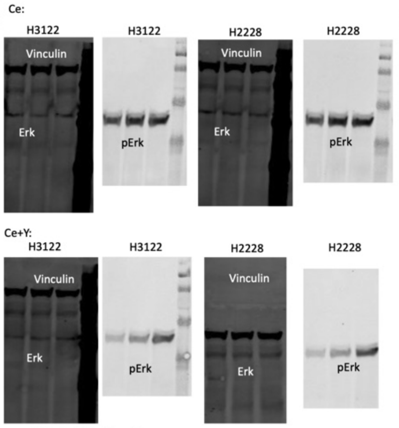

Extended Data Figure 6b

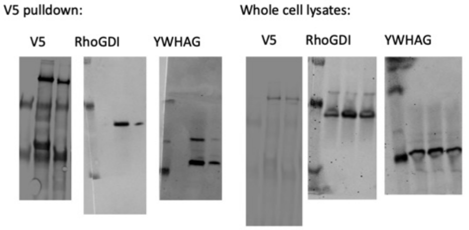

Extended Data Figure 7a

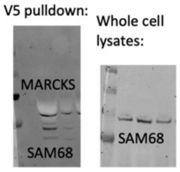

Extended Data Figure 7c

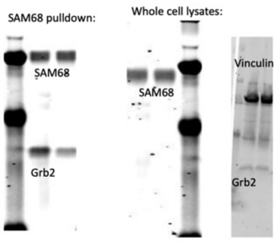

Extended Data Figure 7d

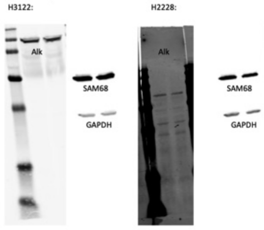

Extended Data Figure 7e:

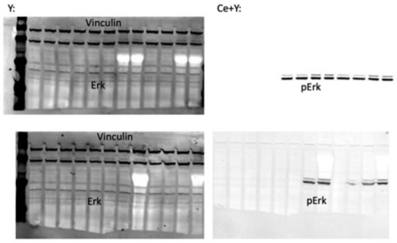

Extended Data Figure 7f:

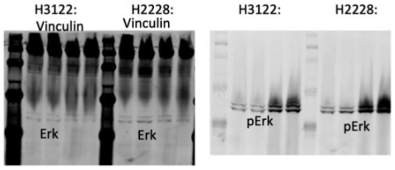

Supplement: Supplementary file 1 — Supplementary Fig. 1 (uncropped gel blots). [file 41587_2023_2107_MOESM1_ESM.pdf]
