# Supplementary material for: Computationally designed sensors detect endogenous Ras activity and signaling effectors at subcellular resolution
Source: Nat Biotechnol. 2024 Jan 25;42(12):1888–98. doi: 10.1038/s41587-023-02107-w (PMC11631767; doi:10.1038/s41587-023-02107-w)
Supplement: Supplementary file 2 — Reporting Summary [file 41587_2023_2107_MOESM2_ESM.pdf]

nature portfolio

Corresponding author(s): Jason Z Zhang, Dustin J Maly, David Baker

Last updated by author(s): 11/27/2023

Reporting Summary

Nature Portfolio wishes to improve the reproducibility of the work that we publish. This form provides structure for consistency and transparency in reporting. For further information on Nature Portfolio policies, see our Editorial Policies and the Editorial Policy Checklist.

Statistics

For all statistical analyses, confirm that the following items are present in the figure legend, table legend, main text, or Methods section.

n/a	Confirmed	
☐	☒	The exact sample size (*n*) for each experimental group/condition, given as a discrete number and unit of measurement
☐	☒	A statement on whether measurements were taken from distinct samples or whether the same sample was measured repeatedly
☐	☒	The statistical test(s) used AND whether they are one- or two-sided<br>*Only common tests should be described solely by name; describe more complex techniques in the Methods section.*
☒	☐	A description of all covariates tested
☒	☐	A description of any assumptions or corrections, such as tests of normality and adjustment for multiple comparisons
☐	☒	A full description of the statistical parameters including central tendency (e.g. means) or other basic estimates (e.g. regression coefficient) AND variation (e.g. standard deviation) or associated estimates of uncertainty (e.g. confidence intervals)
☐	☒	For null hypothesis testing, the test statistic (e.g. *F*, *t*, *r*) with confidence intervals, effect sizes, degrees of freedom and *P* value noted<br>*Give P values as exact values whenever suitable.*
☒	☐	For Bayesian analysis, information on the choice of priors and Markov chain Monte Carlo settings
☒	☐	For hierarchical and complex designs, identification of the appropriate level for tests and full reporting of outcomes
☐	☒	Estimates of effect sizes (e.g. Cohen's *d*, Pearson's *r*), indicating how they were calculated

Our web collection on statistics for biologists contains articles on many of the points above.

Software and code

Policy information about availability of computer code

Data collection	Epiluorescence images were captured through NIS Elements 5.30 software. Some brightfield images were captured on ZOE Fluorescent Cell Imager.
Data analysis	Rosettascripts was used for initial design of Ras-LOCKR proteins. For analyzing raw mass spectrometry data, MaxQuant/Andromeda version 1.5.2.8 was used. Further statistical analysis of mass spectrometry data was processed using Perseus software package v1.5.6.0. Panther database was further used to cluster mass spectrometry hits. Fiji 2.13.1 was used to analyze epifluorescence images. Data was analyzed and graphed on GraphPad Prism 8.

For manuscripts utilizing custom algorithms or software that are central to the research but not yet described in published literature, software must be made available to editors and reviewers. We strongly encourage code deposition in a community repository (e.g. GitHub). See the Nature Portfolio guidelines for submitting code & software for further information.

Data

Policy information about availability of data

All manuscripts must include a data availability statement. This statement should provide the following information, where applicable:

- Accession codes, unique identifiers, or web links for publicly available datasets
- A description of any restrictions on data availability
- For clinical datasets or third party data, please ensure that the statement adheres to our policy

The data that support the findings of this study are available from Figshare (https://figshare.com/projects/Computationally_designed_sensors_detect_Ras_activity_and_signaling_effectors_at_subcellular_resolution/186933)50. Proteomic raw data is available on PRIDE (https://massive.ucsd.edu/ProteoSAFe/dataset.jsp?task=66ee1bb3919049f0a7465bb199e2e324). All accession codes have been provided for the paper.

Human research participants

Policy information about studies involving human research participants and Sex and Gender in Research.

Reporting on sex and gender	N/A
Population characteristics	N/A
Recruitment	N/A
Ethics oversight	N/A

Note that full information on the approval of the study protocol must also be provided in the manuscript.

Field-specific reporting

Please select the one below that is the best fit for your research. If you are not sure, read the appropriate sections before making your selection.

☒ Life sciences ☐ Behavioural & social sciences ☐ Ecological, evolutionary & environmental sciences

For a reference copy of the document with all sections, see nature.com/documents/nr-reporting-summary-flat.pdf

Life sciences study design

All studies must disclose on these points even when the disclosure is negative.

Sample size	All experiments were done in at least triplicate. The sample size is chosen based on sufficiency for statistical analysis for calculating assay specificity and sensitivity.
Data exclusions	No sample was excluded from data analysis.
Replication	The results were successfully replicated using different cell line stocks and on different days. For in vitro experiments, the results were successfully replicated using different batches of pure proteins on different days.
Randomization	Beyond expression of transfected plasmids, selection of cells for fluorescent images was randomized. No further randomization was performed as this is not a treatment/response study.
Blinding	Data collection was not blinded as sample treatment and collection requires prior knowledge of the experiment. Data was first processed in blinded mode, followed by sample/control assignment.

Reporting for specific materials, systems and methods

We require information from authors about some types of materials, experimental systems and methods used in many studies. Here, indicate whether each material, system or method listed is relevant to your study. If you are not sure if a list item applies to your research, read the appropriate section before selecting a response.

Materials & experimental systems

n/a	Involved in the study
☐	☒ Antibodies
☐	☒ Eukaryotic cell lines
☒	☐ Palaeontology and archaeology
☒	☐ Animals and other organisms
☒	☐ Clinical data
☒	☐ Dual use research of concern

Methods

n/a	Involved in the study
☒	☐ ChIP-seq
☒	☐ Flow cytometry
☒	☐ MRI-based neuroimaging

Antibodies

Antibodies used

Antibody dilution factors are listed in Supplementary Table 4.
Rb anti-Alk CST 3633
Ms anti-Erk CST 9107
Rb anti-phospho-Erk CST 9101
Ms anti-Akt CST 2920
Rb anti-phospho-Akt (S473) CST 9271
Rb anti-GAPDH CST 5174
Ms anti-Vinculin Sigma-Aldrich V9131
Ms anti-Myc Tag CST 2276
Ms anti-Flag Tag Sigma-Aldrich A8592
Rb anti-V5 Tag CST 13202
Ms anti-V5 Tag CST 80076
Ms anti-CD3ε Ancell 144-020
Ms anti-CD28 Ancell 177-020
Rb anti-E cadherin Abcam ab40772
Ms anti-Giantin Abcam ab37266
Rb anti-Bcl-xl CST 2764
Ms anti-Bcl-xl Abcam ab77571
Rb anti-pan Ras CST 91054
Rb anti-HA Tag CST 3724
Rb anti-GFP CST 2555
Rb anti-RhoGDI CST 2564
Ms anti-SAM68 Santa Cruz Biotechnology sc-514468
Rb anti-SAM68 CST 33210
Rb anti-YWHAG Abcam ab155050
Rb anti-MARCKS CST 5607
Ms anti-Grb2 Abcam ab281846
Rb anti-Grb2 Abcam ab32037
680 RD anti-Ms LICOR 26-68071
680 RD anti-Rb LICOR 926-68070
800 CW anti-Ms LICOR 926-32210
800 CW anti-Rb LICOR 926-32211
AF488 anti-Ms Thermo Fisher A11029
AF568 anti-Rb Thermo Fisher A11036
AF488 anti-Rb Thermo Fisher A11034
AF750 anti-Ms Thermo Fisher A21037

Validation

Validation of all antibodies are provided on manufacturer's websites for specific application (immunostaining, immunoblotting). All of these antibodies have been used in previous literature as well. Example data is shown on manufacturer's website (e.g. target protein KO).
For all CST primary antibodies listed here, antibodies are validated in gene KO cells siRNA KD cells, heterzygous KO cells, different cell lines, and in other antibody applications such as competitive ELISA, peptide dot blots, peptide blocking, or protein arrays.
For all Sigma-Aldrich primary antibodies listed here, antibodies underwent enhanced validation efforts which include validation in gene KO cells, gene overexpression cells, and orthogonal assays (e.g. RNAseq correlation, Immunocapture-MS).
Abcam antibodies Rb anti-E cadherin Abcam ab40772, Ms anti-Giantin Abcam ab37266, Ms anti-Grb2 Abcam ab281846 were validated in KO cells.
Ms anti-SAM68 Santa Cruz Biotechnology sc-514468 was validated in siRNA KD cells.

Eukaryotic cell lines

Policy information about cell lines and Sex and Gender in Research

Cell line source(s)

MEF KRas4A NCI RPZ26187
MEF KRas4B NCI RPZ25854
MEF HRas NCI RP200024
MEF NRas NCI RPZ26379
MEF WT ATCC CRL-2991
HEK293T ATCC CRL-3216
HEK293F ATCC CRL-1573

	HEK293-FlpIn TRex Invitrogen R78007<br>HeLa ATCC CCL-2<br>Beas2B ATCC CRL-9609<br>Jurkat ATCC TIB-152<br>H3122 Gift from R. Bayliss lab<br>H2228 Gift from R. Bayliss lab
Authentication	Cell lines were authenticated by provider.<br>MEF cells were sequenced and validated for KO via immunoblotting.<br>All ATCC cell lines have undergone STR profiling.<br>H3122 and H2228 (by ATCC) cells have undergone STR profiling.
Mycoplasma contamination	All cell lines were tested every 2 months for mycoplasma contamination via PCR test. None of these cell lines were positive for mycoplasma.
Commonly misidentified lines<br>(See ICLAC register)	None in this study.
